# Differences in nursing home admission between functionally defined populations in Germany and the association with quality of health care

**DOI:** 10.1186/s12913-021-06196-8

**Published:** 2021-03-02

**Authors:** Dominik Domhoff, Kathrin Seibert, Susanne Stiefler, Karin Wolf-Ostermann, Dirk Peschke

**Affiliations:** 1 Institute for Public Health and Nursing Research, Faculty 11: Human and Health Sciences, University of Bremen, Bremen, Germany; 2 High Profile Area Health Sciences, University of Bremen, Bremen, Germany; 3 Department of Applied Health Sciences, Hochschule für Gesundheit (University of Applied Sciences), Bochum, Germany

**Keywords:** Nursing home admission, Care-dependency, Patient-sharing networks, Health care provision, Quality indicators, Health disparities

## Abstract

**Background:**

People prefer to age in place and not move into a nursing home as long as possible. The prevention of cognitive and functional impairments is feasible to support this goal. Health services play a key role in providing support for underlying medical conditions. We examined differentials in nursing home admissions between patient sharing networks in Germany and whether potential variations can be attributed to indicators of health care provision.

**Methods:**

We conducted an ecological study using data of patients of 65 years and above from all 11 AOK statutory health insurance companies in Germany. Nursing home admissions were observed in a cohort of persons becoming initially care-dependent in 2006 (*n* = 118,213) with a follow-up of up to 10 years. A patient sharing network was constructed and indicators for quality of health care were calculated based on data of up to 6.6 million patients per year. Community detection was applied to gain distinct patient populations. Analyses were conducted descriptively and through regression analyses to identify the variation explained by included quality indicators.

**Results:**

The difference in the proportion of nursing home admissions between identified clusters shows an interquartile range (IQR) of 12.6% and the average time between onset of care-dependency and admission to a nursing home an IQR of 10,4 quarters. Included quality indicators attributed for 40% of these variations for the proportion of nursing home admissions and 49% for the time until nursing home admission, respectively. Indicators of process quality showed the single highest contribution. Effects of single indicators were inconclusive.

**Conclusions:**

Health services can support persons in their preference to age in place. Research and discussion on adequate health care for care-dependent persons and on conditions, where nursing home admission may be beneficial, is necessary.

**Supplementary Information:**

The online version contains supplementary material available at 10.1186/s12913-021-06196-8.

## Background

When asked for their housing preferences in old age and in possible need of support peoples preferred place to stay is in their own home. Nursing homes are far less favoured as long as alternatives are feasible [1–4]. Enabling people to age in place is a fundamental societal task, especially calling for adequate engagement of health care services.

Assuming that cognitive and functional impairments, which occur more frequently in old age, are regularly consequences of or accompanied by conditions that require medical treatment, health services play a major role in supporting patients’ preferences for a long life at home [5, 6]. Potential measures can be derived alongside primary, secondary and tertiary prevention. Primarily, conditions causing impairments should be prevented. Secondary, the manifestation of impairments upon occurrence of diseases should be averted. Tertiary, opportunities to reduce or eliminate impairments eventually should be seized [7]. In the case of care-dependent persons, research on care provision is focused on widespread non-communicable diseases like dementia, diabetes or cardiovascular-diseases, where these conditions show inconclusive results in terms of nursing home admissions [6].

Regional variations in German long-term care are well-known with proportions of care-dependent persons being cared for in nursing homes varying between federal states [8]. Albeit, there is only little research on these differentials based on the available long-term care infrastructure [9]. Regional differences in health care provision recently got into focus of research and unwarranted variations proved to be present in Germany [10]. The comparison of distinct populations allows to reveal attainable goals and determining factors associated with the outcomes of interest and consequently identify possibilities for improvement [11].

Assuming that delaying or preventing complications arising from mainly chronic conditions contributes to ongoing residency in place of care-dependent persons [6], the quality of ambulatory primary care is of special interest. For this purpose, a wide range of population based quality indicators (QI) is available to assess the quality of processes and outcomes in healthcare [12, 13].

This study aims to identify the extent to which structures, processes and outcomes of the health care provision for care-dependent persons are associated with nursing home admissions. Findings could then enable health care providers to identify potential determinants to support care-dependent persons in the choice of their preferred place of care.

Administrative levels below state level neither have legislative implications for health care provision [14], nor do their boundaries pose limitations regarding health care utilisation [15]. Therefore, we apply the concept of patient sharing networks (PSN) in this study. PSN are social networks of health care providers with the connections defined by the number of shared patients between them [16]. Due to the freedom of choice of providers in the German statutory health insurance (SHI), a PSN is formed through utilisation of providers by the patients. PSN can be described as a functionally defined population in contrast to geographically defined populations [17].

The advantage of employing PSN is the empirical foundation of the identified populations. With multidisciplinarity being an essential characteristic of health care, research on care provision on population level should also include relevant care providers and disciplines [18]. PSN allow to identify relevant providers for all dimensions of health care by means of commonly treated patients, thus representing the effect of care provision to single patients by multiple providers. Furthermore, they take into account the actual choice of providers by patients themselves and their impact on the construction of the individual care network, regardless of geographic boundaries, which may not pose a relevant factor for the choice of the provider [19].

This study addresses the following research questions: (1) to what extent do differentials in nursing home admissions exist between PSN and (2) are different rates of nursing home admissions associated with characteristics of health care provision?

## Methods

With the level of interest in this study being the population level, this study is conceptualised as an ecological study embedded within a cohort study. The data used originate from German SHI claims data and two different study populations. We follow the STROSA 2 reporting standard [20], specifically developed for analyses of secondary data and their specific requirements for the German health care system.

### Study design

This study aims to examine differentials in nursing home admission and associated factors on population level. Thus, it conforms to the characteristics of an ecological study with the outcome variables being determined within a cohort study and the predicting variables as a time series of cross-sectional observations. Analyses were conducted descriptively and by multivariate regression analyses.

### Data source

To answer these questions, we use German SHI claims data. Due to their completeness regarding health care service utilisation, they allow the construction of PSN, the evaluation of relevant quality indicators for health care provision and contain information on the type of services received from the statutory long-term care insurance.

The study is based on claims data from persons aged 65 year or older, insured at one of the 11 AOK statutory health insurance companies in Germany. Available data comprises the entire individual in- and outpatient health care history of the study participants, including all diagnoses, prescribed and provided medications, medical procedures, rehabilitation services and physical, occupational, speech and language therapy, podology, and care status and services (nursing home or home care) as well as personal data including age, sex, and federal state.

### Sample and sample size

The primary study cohort comprised persons aged 65 years and above having become care-dependent according to the 11th Book of the German Social Code for the first time in 2006 and insured at an AOK SHI company (*n* = 118,213). There were no further inclusion criteria. Persons were excluded from the study cohort when they were admitted to a nursing home in 2006, as it was not possible to determine temporal order and interval between assessment of care dependency and nursing home admission for this year. Individuals in the study cohort were followed up until they met the target event of nursing home admission, deceased or changed their insurance company. Observational period lasted no longer than the year 2016.

The network dataset comprised all persons aged 65 years and above, who utilised services from any provider who provided services to any patient of the study cohort. Persons were included on a quarterly basis according to these criteria. Consequently, the resulting dataset contained between 5.8 and 6.6 million persons per year.

Due to the exploratory nature of the analyses and the lack of defined intervention groups, no ex-ante sample size calculation was conducted.

### Data protection

Data was provided by the AOK Federal Association as the data holder on behalf of the AOK insurance companies as data owners. The data holder anonymised the data to preclude the identification of individuals while retaining the possibility to observe individuals longitudinally and between sectors of health care. The data protection officer of the AOK Federal Association approved an operation procedure for data protection, restricting usage of the data for the studies purpose.

Datasets containing information of the study cohort were provided to the members of the research project. For the network dataset only, a sample was provided to the study team, analyses on the full dataset were conducted within the facilities of the data holder.

### Network construction

With the comparison of populations as the primary characteristic of an ecological study, the level of aggregation is a key aspect. This study uses an empirically determined approach to identify functionally defined populations of service providers and their respective patients. Therefore, we employ the concept of PSN, where care providers are connected to each other through commonly treated patients [16, 21–23]. In terms of social network analysis, PSN establish networks with care providers as nodes and the patients treated by any dyad of providers as edges.

Based on the network dataset we constructed a PSN including providers of all sectors identified by their site identification number (BSNR) from 2006 to 2016 as nodes (*n* = 333,859). We excluded anaesthetists, laboratory medicine, pathologists, radiologists, and nuclear medicine from the network, as we do not assume a strong personal contact between patients and members of these disciplines as well as a reduced freedom of choice of single providers from these specialties. Edges (*n* = 24,836,924) between two providers are present, when there was at least one patient treated by the two connected providers. The number of patients that two providers had in common were assigned to the edges as their weight.

As a network constructed in such a manner does not inherently allow distinguishing groups of actors, a clustering or community detection algorithm has to be applied. We used the Speaker-listener Label Propagation Algorithm (SLPA) [24] which allows the processing of weighted and directed network graphs as well as overlapping nodes between clusters. The latter is especially important as patients in the German SHI can freely choose their providers. While there is a large heterogeneity of disciplines present in the PSN, it cannot be foreclosed that single providers are relevant members in more than one cluster.

To reduce complexity of the network graph, we converted the graph from an undirected to a directed graph by duplicating the edges. Subsequently, only the 20% strongest outgoing edges to other providers were retained. This procedure takes into account, that the strength of connections to other actors varies with the total number of patients served and thus differs largely between sectors, disciplines and providers.

SLPA identified 419 clusters in the PSN. These contained on average 805 providers (Q1: 261; Q3: 806). One node belonged to 1.01 clusters on average and to 4 clusters at the maximum (1:n relation), which indicates a low overlap between clusters. Subsequently, patients from the study cohort and network dataset were assigned to the identified clusters. Therefore, patients were on a yearly basis assigned to the cluster that provided the single most cases of treatment during 1 y. On average, this primary cluster was responsible for 90.3% of all cases of treatment and 93.3% of all visits of the individual patients.

### Variables

Aiming to describe the differences in nursing home admissions in different populations, there are two outcome variables of interest: (1) proportion of care-dependent persons being admitted to nursing homes, and (2) the duration between onset of care-dependency and the admission to a nursing home. Existing systematic reviews on nursing home admissions primarily found hazard ratios from survival analysis [5, 25]. These “account not only for the number of events but also for the timing when these events occur “[25]. As this study does not allow estimating hazard ratios due to its design, we derived the two outcomes variables stated above to depict both dimensions. As availability of nursing homes differs regionally [9] and affects whether a placement can take place, it is expected that both outcome variables have different underlying processes. Both outcome variables were derived from the study cohort through aggregation.

Explanatory variables consist of indicators on the health services provision as well as compositional attributes of the identified clusters. To address characteristics of health care provision, we identified established population-based QI for health care based on a Systematic Review [26]. Indicators used in the studies were extracted and feasibility for the study was assessed by the study team based on the available SHI data. Included were 68 QI, that could be calculated with the data available in the claims, from 13 areas: Ambulatory care sensitive cases (1 QI), asthma (4 QI; prevalence, performed spirometry, medication), chronic obstructive pulmonary disease (COPD; 8 QI; e.g. prevalence, medication, acute inpatient treatment, respiratory therapy), cardiovascular diseases (CVD; 14 QI; e.g. prevalences, medication, acute inpatient-treatment, performed electrocardiogram), dementia (2 QI; prevalence and blood analysis), depression (2 QI; prevalence and pharmacotherapy), medication (7 QI; potential inadequate medication for the elderly, polypharmacy and medication on certain conditions), osteoarthritis (1 QI; prevalence), osteoporosis (1 QI; prevalence), prevention (5 QI; cancer screening, vaccination), type 2 diabetes mellitus (T2D) (7 QI; prevalence, blood analysis, ophthalmological examinations, comorbidities), number of comorbidities (1 QI) and continuity of care for selected conditions (15 QI; Continuity of Care Index, Sequential Continuity Index, Usual Provider Continuity Index). These could be grouped into 4 overarching categories: Morbidity indicators (10 QI), care process indicators (40 QI), outcome indicators (3 QI) and indicators for continuity of care (15 QI). Additionally, we included indicators on the structure of the clusters with the proportions of 12 single medical specialities and sectors (hospitals, physical, occupational and speech therapists and podologists) on all providers in the cluster, the absolute number of providers in the cluster and the proportion of patients in the cluster being care-dependent. A full description of the indicators and their calculation can be found in Additional File 1.

### Data processing

For the outcome variables, we identified the last observed quarter of each person within the study cohort and thereby measured the time between onset of care dependency and their institutionalisation. As we could not determine the exact quarter of the onset of care-dependency in 2006, we assumed the beginning of care-dependency to be in the fourth quarter of 2006. Through aggregation and standardisation, we obtained the average time in quarters between onset of care-dependency and the proportion of persons admitted to a nursing home in the follow-up for each cluster, standardised by age and sex. For standardisation, we used the new standard population of Europe (1990) [27].

The explanatory variables were calculated per cluster on a quarterly basis and also standardized by age and sex. Due to a distinct observed increasing trend in most indicators over time, we performed detrending of these time series. For each indicator a linear regression for all clusters was estimated with the indicator value as the dependent and the consecutive number of the quarter as the independent variable. The resulting average slope was then subtracted from the time series of each cluster. That way we took into account the relative ordinance between and individual performance of clusters and seasonality, while we excluded overarching trends that affect the entire study cohort and may correlate with a later institutionalisation.

Subsequently, time series of each cluster where aggregated into one observation per cluster. Each quarter went into calculation of the average for each cluster weighted by the number of persons assigned in that quarter. In this way the impact of outliers is reduced and performance of a cluster is deemed more important when it supplies services for a larger number of persons.

Provider composition of clusters was only calculated once and remained constant, as the clusters in our model were constructed based on the entire observational period. All figures representing proportions were multiplied by 100 to receive meaningful regression coefficients which can be interpreted as 1 % change.

### Statistical analyses

Initially, we descriptively analyse and describe the characteristics of the study cohort, that the outcome variables are based on, and the resulting aggregated dataset. The relationship between the two outcome variables is examined further to identify existing patterns in nursing home admissions. As evidence for nursing home admission on population level is rare for Germany, this may provide further insight on processes leading to nursing home admissions.

To assess characteristics potentially associated with nursing home admission and their contribution towards statistically explaining differentials, we estimated hierarchical Ordinary Least Squares (OLS) regression models, with dependent and independent variables being an interval scale. Prior to regression analysis, we calculated Pearson correlations for all pairs of independent variables included to assess possible collinearity between variables. For each outcome variable, we estimated one regression model per indicator category, one model including all explanatory variables and one model applying backwards elimination to the model containing all variables. We set the level of significance to 0.05 and report 95% confidence intervals. To minimize the potential impact of extreme values from clusters with only a small sample size, we carried out the regression weighted by the average number of patients from the study cohort assigned to a cluster. Formula 1 presents the regression equation with *β*_0_ being the constant and *β*_1_ – *β*_*j*_ the regression coefficients to be determined, *j* representing the independent variables in the model and *i* the clusters, thus *x*_*ij*_ being the value of each independent variable for each cluster and *e* being the residual. The indicators included in each model specification are listed in Additional File 2.

1 $$ {y}_i={\beta}_0+{\beta}_1{x}_{i1}+\dots +{\beta}_j{x}_{ij}+{e}_i $$

We assume that residuals are normally distributed and have a smaller variance in larger clusters, where *e*_*i*_~*N*(0, *σ*^2^/*w*_*i*_) with *w*_*i*_ being the average number of patients assigned to a cluster. Weighted least squares regression minimises the weighted residual sum of squares (WRSS) [28] as depicted in Formula 2, with *n* being the number of observed clusters.

2 $$ WRSS\left({\beta}_0,{\beta}_1\dots {\beta}_j\right)=\sum \limits_{\mathrm{i}=1}^n{w}_i{\left({y}_i-{\beta}_0-{\beta}_1{x}_{i1}-\dots -{\beta}_j{x}_{ij}\right)}^2 $$

As single indicators are not of primary interest in this analyses, we only report the types of covariates included in the regression models in the main results alongside with R^2^, Adjusted R^2^ [29] and Akaikes Information Criterion (AIC) as measure for goodness of fit [30]. Data preparation was conducted in SAS 9.4 [31], descriptive and regression analyses were performed in R 3.6.1 [32].

## Results

Table 1 shows the main characteristics of the study cohort. During the 10-year follow-up 28.3% of the care-dependent persons were admitted to a nursing home. These individuals were more likely to be female, on average older and had a lower nursing care level upon becoming initially care dependent than those who never lived in an institution. Follow up for persons in home care was longer but shows a large variation for both subgroups (Table 1).

**Table 1:** Description of study cohort according to 2007, 1st quarter (*n* = 118,213)

	Without admission to nursing home (***n*** = 84,759)	With admission to nursing home (***n*** = 33,454)
**Sex**		
Male	30,663 (36.2%)	8193 (24.5%)
Female	54,096 (63.8%)	25,261 (75.5%)
**Mean age in years (SD)**	81.1 (7.3)	83.1 (6.9)
**Nursing care level**		
1	61,038 (72.0%)	26,417 (79.0%)
2	19,798 (23.4%)	6198 (18.5%)
3	3190 (3.8%)	532 (1.6%)
none	733 (0.9%)	307 (0.9%)
**Follow-up in quarters (SD)**	15.8 (12.7)	9.1 (8.5)

Using SLPA for community detection, 419 provider clusters were identified in the network data set. Patients from the study cohort were assigned to 407 out of 419 clusters. No patient obtained a majority of health services from one of the remaining 12 clusters, whereby no indicators could be calculated for these clusters. The main characteristics of the clusters after aggregation are presented in Table 2. Sizes of the clusters determined through the number of included providers, patients from the study cohort and the network dataset varied highly between clusters. Description of all included variables is supplied in Additional File 3.

**Table 2:** Description of provider cluster attributes (*n* = 407)

Attributes	Mean (SD)	Median (Q1, Q3)
**Quarters until admission to nursing home**	13.4 (7.0)	13.9 (7.8, 18.2)
**Percent of care-dependent persons admitted to nursing home**	22.5 (10.5)	22.1 (16.2, 28.8)
**No. of Comorbidities of all patients in clusters**	1.9 (0.2)	1.8 (1.7, 2.0)
**No. of persons from study cohort in clusters**	290.4 (376.5)	183.0 (84.5, 361.0)
**No. of persons treated by clusters**	17,075.4 (19,340.6)	11,120.6 (5898.8, 21,773.0)
**No. of providers in cluster**	819.8 (980.4)	499.0 (273.0, 998.5)

*SD* standard deviation, *Q1* first quartile (25th percentile), *Q3* third quartile (75th percentile); clusters constructed using SLPA [24]

**Fig. 1:**
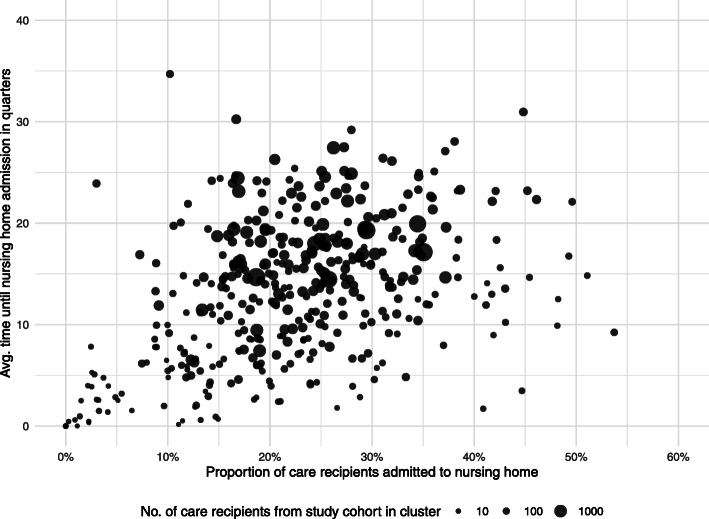
Distribution of nursing home admission rate and average time until institutionalisation by cluster. Legend: Standardised by age and sex; *n* = 407

Figure 1 plots the proportion of care-dependent persons admitted to a nursing home against the average time between onset of care-dependency and admission to a nursing home for each cluster based on the study cohort. While the mean proportion of persons being admitted to a nursing home is 22.5%, this event appears on average 13.4 quarters after the acknowledgement of care-dependency. Dividing all clusters into tertiles, the lowest tertile shows an average share of 11.5% of the study cohort being admitted to a nursing home in the follow up with the medium and upper tertiles showing figures of 22.3 and 33.9%. The average time until an observed institutionalisation ranges from 5.4 quarters in the lower, to 13.7 quarters in the medium and 21.0 quarters in the upper tertile.

Both variables present a moderate correlation (r = 0.44, 95% CI 0.35–0.51) between each other. Taking into account the number of patients from the study cohort, the differences in both variables are not primarily caused by smaller clusters. However, the lower half of the average time interval seems to be dominated by clusters with a smaller amount of persons from the study cohort (Fig. 1).

Table 3 shows the results from the regression analyses for the proportion of nursing home admissions as the outcome variable. With all explanatory variables included (Model 6), 36% of the variance between clusters could be statistically explained according to the Adjusted R^2^, which – contrary to R^2^ – includes a penalisation for the number of explanatory variables. The single largest contribution towards this originates from the dimension of care process indicators (Model 3), which could explain 26% of the variation, followed by indicators for morbidity (Model 2) and continuity of care (Model 4) with 18% each. Composition of the clusters (Model 1) and outcome indicators (Model 5) play a less important role in explaining variance. Applying backwards elimination, only 34 of all 82 explanatory variables from all dimensions remain in Model 7, with R^2^ declining by 3 percentage points and Adjusted R^2^ increasing by 4 percentage points due to less penalisation for the number of variables.

**Table 3:** Proportion of nursing home admissions: variance explained by dimensions of health care provision and morbidity

Variables included	Model 1	Model 2	Model 3	Model 4	Model 5	Model 6	Model 7	
**Composition**	x					x	Backward elimination	x
**Morbidity**		x				x		x
**Process indicators**			x			x		x
**Continuity of Care**				x		x		x
**Outcome indicators**					x	x		x
**No. of variables**	14	10	40	15	3	82	34	
**No. obs.**	407	407	407	407	407	407	407	
**R**^**2**^	0.11	0.20	0.33	0.21	0.02	0.49	0.46	
**Adj. R**^**2**^	0.08	0.18	0.26	0.18	0.01	0.36	0.40	
**AIC**	3058	3010	2996	3013	3078	2967	2912	

Examining the single coefficients in Additional File 4, only few indicators show significant associations with the proportion of nursing home admissions and if so, the directions of effect are ambiguous. Indicators that are supposed to be undesirable, like the amount of persons with potentially inappropriate medications from the PRISCUS list [33], show an association that leads to a lower number of institutionalisations. Although Model 6 includes a large number of variables, it shows the best fit according to AIC before backward elimination.

Regression analyses using the same explanatory variables were conducted for the time between onset of care dependency and nursing home admission. Results are presented in Table 4. The model including all variables (Model 6) reaches an Adjusted R^2^ of 45% and exceeds the variance explained on the proportion of nursing home admissions by 9 percentage points. While indicators of process quality (Model 3) remain the dimension with the largest contribution towards this, for the time interval we see the composition of the clusters (Model 1) resolving one fourth of the variations between clusters. Morbidity (Model 2) explains less variance of the differentials in time to nursing home admission than for differentials in admission rate. The backward elimination in Model 7 again leads to 34 included variables, partially differing from the respective model of the prior outcome variable, a slight decline of R^2^ and an increase of Adjusted R^2^.

**Table 4:** Average time until nursing home admission: variance explained by dimensions of health care provision and morbidity

Variables included	Model 1	Model 2	Model 3	Model 4	Model 5	Model 6	Model 7	
**Composition**	x					x	Backward elimination	x
**Morbidity**		x				x		x
**Process indicators**			x			x		x
**Continuity of Care**				x		x		x
**Outcome indicators**					x	x		x
**No. of variables**	14	10	40	15	3	82	34	
**Num. obs.**	407	407	407	407	407	407	407	
**R2**	0.28	0.11	0.38	0.16	0.00	0.56	0.53	
**Adj. R2**	0.25	0.09	0.32	0.13	0.00	0.45	0.49	
**AIC**	2697	2776	2686	2759	2807	2634	2563	

Single coefficients in Additional File 5 again show inconclusive results. Favourable quality indicator manifestations do not necessarily correspond to a prolonged living at home and vice versa. For both outcome variables we see more favourable outcomes alongside a higher proportion of patients in a cluster being care-dependent. An increase in the proportion of care-dependent persons by one percentage point comes with a reduction of one percentage point of care-dependent persons admitted to a nursing home and half a quarter delayed admission to a nursing home.

## Discussion

The presented results show pronounced differentials in nursing home admissions of persons being care-dependent according to the 11th Book of the German Social Code in Germany when comparing admission rates and duration until admission between clusters of health care providers. The proportion of care-dependent persons being admitted to a nursing home as well as the time between the initial onset of care-dependency were at least three times as high in the upper than in the lower tertile of clusters. Also, time until nursing home admissions and proportion of care-dependent persons admitted to a nursing home were not unanimously linked to each other, showing diverse patterns of nursing home admissions in the clusters. For both aspects of nursing home admittance, the examined determinants of health care provision did contribute to a significant proportion of the observed variance between the clusters of 40 and 49%, respectively. This underlines that the provision of health services may play an important role in the process leading to a possible institutionalisation of care-dependent persons. Assuming that structures and processes in health care are potentially influenceable, providers are therefore able to support the preferences of older and care-dependent persons to stay at home as long as possible [1–4].

The observed differences in admissions to inpatient long-term care between populations confirm existing results on regional variations in the proportions of care-dependent persons living in a nursing home [8]. Even though the age distribution is seen as one of the origins of those differences [34], the differences still remained after standardisation for age and sex in our analysis. Besides only few existing studies on care trajectories of care-dependent persons, there is, to our knowledge, no existing evidence on the time between onset of care-dependency and the potential admission to a nursing home and its influencing factors on a contextual level until now. Nevertheless, with the knowledge, that people’s desire is to “stay at home as long as possible”, we see this as a relevant outcome to examine, besides the more extensively researched individual sociodemographic and disease-specific factors [35].

Included dimensions of health care delivery proved to be associated with nursing home admission while also accounting for a considerable amount of the observed differentials between clusters. Indicators of process quality of health care statistically explained most of the variations between clusters for both outcome variables, which may be caused by the majority of quality indicators proposed in the literature originating from this dimension. Predominantly, indicators were highly specific regarding the underlying conditions and as such, would not necessarily affect every care-dependent person included in this study. Rather, fulfilment of established indicators of health care quality and following medical guidelines were considered as context indicators for the overall quality of care delivery. In the light of the number of included indicators and the ambiguous results for the single indicators this seems even more relevant.

Indicators for morbidity and the continuity of care were more relevant regarding the proportion of care-dependent persons admitted to a nursing home than for the time until this event occurs. For the latter, the composition of the constructed clusters explained a considerable amount of variation. Thus, accessibility and continuity of care in general practice and specialist care should be ensured for care-dependent persons.

As a single predictor, the proportion of care-dependent persons in a cluster presented a stable association with both outcomes examined. This indicates a volume-outcome-relationship where caring for a larger number of care-dependent persons leads to beneficial outcomes in terms of nursing home admission. Such volume-outcome relationships are usually discussed in the context of better outcomes of surgical procedures in hospitals [36]. There is also evidence of comparable effects for medical care for people with dementia in general practice [37] and from long-term care [38], with higher numbers of people being cared for resulting in better outcomes. To our knowledge, there is no specific evidence yet on volume-outcome relationships for care-dependent people in primary care. While concentration of specific procedures is discussed for inpatient care, this approach does not seem reasonable for conditions common in primary care. Concepts entailing coordination of care and advancing services by training with special respect to care-dependent persons appear more suitable.

From the systematic research for quality indicators only three indicators for outcome quality were included in the analyses and did not account for a considerable amount of the variations between clusters. Due to the small number of indicators identified in this dimension, all relating to acute admissions to hospital in combination with specific conditions, we cannot conclude in how far health-related outcomes are associated with nursing home admissions or whether these constitute two mainly independent outcomes.

With less than half of the included indicators considerably contributing towards explaining the variance in nursing home admission between clusters, the clinical relevance of the single indicators for health care quality for care-dependent persons should be discussed. With some favourable indicators of process quality and especially continuity of care leading to adverse effects in nursing home admissions, oversupply may pose a possible issue for care-dependent persons, where medically indicated behaviour may counteract the individual’s preference for a continued living at home. Likewise, inconclusive effects of specific diseases on nursing home admissions have already been reported before [6]. For further insights, overarching dimensions in health care provision have to be identified, that focus on the needs and peculiarities of care-dependent persons. Such studies should also take into account the presence of illness, medical evidence and patient preference [11] especially for care-dependent patients. While for designated conditions a measurable guideline adherence is viable and desirable, this may not necessarily hold true for multimorbid [39, 40] or care-dependent [41–44] populations. Consequently, further research on the processes and determinants of health care for care-dependent persons is necessary and should be advanced. In addition, the question may arise, under which health conditions and individual circumstances the admission to a nursing home may be desirable from an individual and societal perspective.

### Limitations

Beyond the examined indicators of health care provision, known determinants for nursing home admission on individual level [35] could not be considered in this study. This especially includes cognitive and functional limitations, external factors like family care and other social support or the availability and cost of nursing homes [6]. Regional differences in the inpatient care ratio were described before and primarily related to the local supply of nursing homes [9]. As individual care services and facilities were not available in the data, we could not include these in the PSN.

Further limitations of this study have to be considered. With an ecological study design, we cannot establish causal relationships between the examined indicators and individual determinants leading to a nursing home admission. With the comparison between populations, we aimed to describe differentials for identifying potentials for improvements on a population level. In combination with indicators conceptualised for describing health care provision on population level, this study type is coherent with the research questions. The usage of a cohort study with defined inclusion and exclusion criteria and follow-up allowed to validly determine the outcome variables.

From a methodological point of view, it cannot be proven, that the observed differentials originate from the clusters themselves. With a high geographical proximity of providers within a cluster being present, clusters with comparable outcomes for nursing home admissions may also be spatially adjacent. Actual reasons for the observed differentials between clusters may originate from higher-level regional processes, like state-level regulation. Also, we did not adjust for further geographic factors in this study due to a lack of spatial information for included providers and patients. With ample evidence on regional disparities in terms of structures, processes and outcomes [45], variations in nursing home admission between clusters could be accountable to factors not included in this study. Our approach of using clusters within a PSN was especially chosen to reduce the artefacts that are introduced by choosing arbitrary administrative regions for population-based analyses [46]. With an observed small amount of overlap of clusters, we would expect only a small amount of spatial autocorrelation in the indicators of health care quality. Additionally, the provider clusters cannot be validated regarding their factual meaning for health care provision. Considering the average size of the clusters it seems improbable that there is cooperation between or even knowledge of all providers in the cluster. Different clustering algorithms may therefore lead to diverging results. However, the usage of PSNs should lead to clusters of providers that are actually working with the same patients by taking into account the patients’ freedom of choice of providers in the German SHI.

The number of analyses conducted in combination with the number of indicators included may also have introduced false-positive coefficients in the regression analyses. We did not adjust for multiple testing, as the single coefficients were not of interest due to our research questions and due the lack of a single explanatory variable of interest. Furthermore, overfitting [47] has to be considered for the regression models containing all variables. This could lead to misleading high values of R^2^ and invalid regression coefficients. To counteract the reporting of excessively high coefficients of determination, we primarily reported the Adjusted R^2^ [29], which penalises for the amount of variables included. Furthermore, we calculated correlation coefficients for all pairs of independent variables before regression analyses. We found indicators for continuity of care showing correlations of moderate to high degree and we could not foreclose multicollinearity. This could lead to effects of one independent variable being masked by other included variables [48]. Therefore, we estimated a regression model with all variables included and performed backwards elimination to reduce the number of independent variables while enhancing model fit. Comparing regression coefficients between the model with all variables and the model with backward selection, we do not see large deviations in regression coefficients, which would be symptomatical for multicollinearity [49]. Eventually, interpretation of single regression coefficients is not essential in terms of the research questions.

The study was conducted under the assumption, that indicators of the health care provision of a larger population are associated with the individual process of the admission to a nursing home. With the application of all included population-based indicators to the study cohort, indicators were also applied for conditions that were not necessarily present in the care-dependent individuals. As proxy indicators, these pose the opportunity to promote high quality health care provision in the clusters for the benefit of all patients cared for.

## Conclusions

Considerable differentials between populations in nursing home admissions are present in Germany. More than one third of the variations can be accounted to structures and processes of health care provision, thus proving potential for the advancement of specialised health care for care-dependent persons to provide needs-based health care to support people’s preference to stay at home as long as possible. To support this, policies should focus on further research to identify resources for providing needs-based health care for care-dependent persons and establish guidelines for primary care for care-dependents to ensure equal access to medical care. Among health care providers, awareness needs to be raised on their role in providing medical care for care-dependent persons and their possibly specific needs. Discussion on feasible indicators of health care quality and their target values for care-dependent persons is needed, as well as on strategies for attaining care coordination for this specific group. Nevertheless, conditions where nursing home admissions may be beneficial should also stay in the focus of research.

## Supplementary Information

**Additional file 1.**

**Additional file 2.**

**Additional file 3.**

**Additional file 4.**

**Additional file 5.**

## Data Availability

The use of personal data is restricted by the German Federal Data Protection Act and the EU General Data Protection Act. The respective data holders permitted the usage of the data to the conducting institutions for the scope and period of the study. Data access can only be obtained through data holders.

## Abbreviations

AIC: Akaikes Information Criterion
BSNR: Physician Site Identification Number
COPD: Chronic Obstructive Pulmonary Disease
GP: General Practitioner
HR: Hazard Ratios
IQR: Interquartile Range
NHA: Nursing Home Admissions
OLS: Ordinary Least Squares
PN: Provider Network
PSN: Patient Sharing Network
Q1: First Quartile
Q3: Third Quartile
QI: Quality Indicator
QoC: Quality of Care
R2: Coefficient of Determination
SHI: Statutory Health Insurance
SLPA: Speaker-listener Label Propagation Algorithm
STROSA: German Reporting Standard for Secondary Data Analyses
T2D: Type 2 Diabetes Mellitus
