# Supplementary material for: Differences in nursing home admission between functionally defined populations in Germany and the association with quality of health care
Source: BMC Health Serv Res. 2021 Mar 2;21:190. doi: 10.1186/s12913-021-06196-8 (PMC7923327; doi:10.1186/s12913-021-06196-8)
Supplement: Supplementary file 1 — Additional file 1. [file 12913_2021_6196_MOESM1_ESM.html]

# Definition of the included indicators of health care quality

|  |  |  |  |  |
| --- | --- | --- | --- | --- |
| **Short name** | **Dimension** | **Numerator** | **Denominator** | **Codes used** |
| Composition: General practitioners | Composition | General practitioners | All providers | **FAGR**: 1, 2 |
| Composition: Ophthalmologists | Composition | Ophthalmologists | All providers | **FAGR**: 5 |
| Composition: Surgeons | Composition | Surgeons | All providers | **FAGR**: 6–9, 11, 13, 14 |
| Composition: Multidisciplinary practices | Composition | Multidisciplinary practices | All providers | flagged by data provider |
| Composition: Therapists | Composition | Therapists | All providers | resulting from originating dataset |
| Composition: Internists | Composition | Internists | All providers | **FAGR**: 3, 23–33 |
| Composition: Orthopaedics | Composition | Orthopaedics | All providers | **FAGR**: 10, 12 |
| Composition: Psychologists and psychotherapists | Composition | Psychologists and psychotherapists | All providers | **FAGR**: 44, 47, 51, 53, 58–61, 68, 69 |
| Composition: Other disciplines | Composition | All outpatient providers – outpatient providers in other categories | All providers |  |
| Composition: Urologists | Composition | Urologists | All providers | **FAGR**: 67 |
| Composition: Rehabilitation facilities | Composition | Rehabilitation facilities | All providers | resulting from originating dataset |
| Composition: Hospitals | Composition | Hospitals | All providers | resulting from originating dataset |
| Composition: Proportion of care-dependent persons in cluster | Composition | Persons with approved care-dependency according to 11th Book of the German Social Code assigned to cluster | All persons assigned to cluster |  |
| Composition: Number of providers in cluster | Composition |  |  |  |
| Composition: Number of patients in cluster (network dataset) | Composition |  |  |  |
| Composition: Number of patients in cluster (cohort dataset) | Composition |  |  |  |
| Average number of comorbidities | Morbidity | Number of comorbidities from selected diagnoses | | **ICD-10-GM:** J45.- (Asthma); J43.-, J44.- (COPD); I10–I15 (Hypertension); I50.1 (Heart Failure); I25.- (Coronary Heart Disease); I61.- I63.- I64.- (Apoplexy); F00–F03 (Dementia); E11.- (Type 2 Diabetes mellitus); M15–M19 (Osteoarthritis); M80–M82 (Osteoporosis); F32.- F33.- F34.1 (Depression) |
| COC Asthma | Continuity of Care | Continuity of Care Index for persons with index disease and >= 2 visits to selected providers, describes the contribution of care to the person between providers. 0=each visit to a different provider, 1= all visits to the same provider | | **ICD-10-GM**: J45.- |
| COC COPD | Continuity of Care |  |  | **ICD-10-GM**: J43.- J44.- |
| COC Dementia | Continuity of Care |  |  | **ICD-10-GM**: F00.- F01.- F02.- F03.- |
| COC Diabetes | Continuity of Care |  |  | **ICD-10-GM**: E11.- |
| COC Heart Failure | Continuity of Care |  |  | **ICD-10-GM**: I50.1 |
| SECON Asthma | Continuity of Care | Sequential Continuity Index for persons with index disease and >= 2 visits to selected providers; describes the ratio of sequential pairs of visits to the same provider. 0=each visit to a different provider that the prior visit, 1=all follow-up visits made to the same provider. | | **ICD-10-GM**: J45.- |
| SECON COPD | Continuity of Care |  |  | **ICD-10-GM**: J43.- J44.- |
| SECON Dementia | Continuity of Care |  |  | **ICD-10-GM**: F00.- F01.- F02.- F03.- |
| SECON Diabetes | Continuity of Care |  |  | **ICD-10-GM**: E11.- |
| SECON Heart Failure | Continuity of Care |  |  | **ICD-10-GM**: I50.1 |
| UPC Asthma | Continuity of Care | Usual Provider Continuity Index for persons with index disease and >= 2 visits to selected providers, describes the density of care made by a primary provider. 0=every visit to a different provider, 1=all visits to the same provider. | | **ICD-10-GM**: J45.- |
| UPC COPD | Continuity of Care |  |  | **ICD-10-GM**: J43.- J44.- |
| UPC Dementia | Continuity of Care |  |  | **ICD-10-GM**: F00.- F01.- F02.- F03.- |
| UPC Diabetes | Continuity of Care |  |  | **ICD-10-GM**: E11.- |
| UPC Heart Failure | Continuity of Care |  |  | **ICD-10-GM**: I50.1 |
| Asthma: Prevalence | Morbidity | Persons with bronchial asthma | All persons | **ICD-10-GM**: J45.- |
| Asthma: Spirometry | Outcome indicators | Persons with bronchial asthma and spirometry | Initially diagnosed asthmatics | **ICD-10-GM**: J45.-, no such diagnosis in the prior data year. **GONR**: 03330 04330 04419 04534 13255 13560 13660 27330 04530 13650 36881 **OPS**: 1719 1712 (spirometry) |
| Asthma: Inhalative medication | Process indicators | inhalative medication | Asthmatics with medication | **ICD-10-GM**: J45.-. **ATC**: R03AC02 R03AC03 R03AC04 R03BB01 R03BA R03AC12 R03AC13(inhalative medication); R03DA04 R03CC02 R03CC03 R03CC04 R03CC14 H02AB06 H02AB07 H02AB04 H02AB03 R03DC03 R03AK07 R03AK06 H02AB03 H02AB04 H02AB06 H02AB07 R03DX05 R03DA04 R03CC (other medication); |
| Asthma: ICS | Process indicators | long-term medication of inhalative corticosteroids | Asthmatics with long-term medication | **ICD-10-GM**: J45.-; **ATC**: R03BA (ICS); R03AC12 R03AC13 R03DC03 R03AK07 R03AK06 H02AB03 H02AB04 H02AB06 H02AB07 R03DX05 R03DA04 R03CC (other long-term medication) |
| Medication: PRISCUS | Process indicators | Persons over 65 years with medication from PRISCUS list | Persons over 65 years | **ATC**: C01EB03M01AB01 M01AB51 M02AA23 M02AA73 S01BC01 M01AB11 M01AE03 M02AA10 M01AE53 M01AA01 M02AA01 M01AA51 R05XA10 M01BA01 M01AC01 M02AA07 S01BC06 M01AC06 M01AC56 M01AH05 (NSAID). N02AB02 N02AB52 N02AB72 N02AG03 (opiod analgetics); C08DA81 C01BA01 C01BA51 C01BA71 C01BC04 C07AA07 C07AA57 C07BA07 C01AA05 C01AA55 C01AA02 C01AA52 C01AA08 C01AA58 (antiarrythmic agents); J01XE01 J01XE51 (antibiotics); A03AA A03AB A03CA A03DA A03E N04A R03BB S01FA R03AL (anticholinergic agents); R06AB04 R06AB54 R06AX32 (if year <2015) R06AX07 R06AX57 G04BD04 G04BD04 G04BD07 D04AA14 R06AA04 R06AA54 N05BB01 N05BB51 R06AX33 D04AA13 R01AC09 R06AB03 R01AC08 N05BB52 (antihistamines); B01AC05 B01AC22 (anticoagulants); N06AA09 N06CA01 N06AA12 N06AA02 N06AA04 N06AA21 N06AA06 (antidepressives); N06AB03 (SSRI): N06CA03 N06CA07 N06AF04 (MAO-inhibitors). A04AB02 A04AB52 (antiemetics); C02CA04 G04CA05 C02CA01 C02LE01 C02CA08 G04CA03 (alpha-blockers); C02AC01 N02CX02 N07BB06 S01EA04 C02LC01 C02LC51 C02AA02 N05AX15 N05AX16 C02AA52 C02LA01 C02LA51 C02LA71 C02AB01 C02LB01 C02AB02 (cardio-vascular medication); C08CA05 (calcium-channel blockers); N05AC02 N05AB02 N05AA02 N05AB03 N05AD01 N05AH03 N05AH02 (neuroleptics); N02CA01 N02CA51 N02CA71 C06AA02 C06AA50 N02CA02 N02CA52 N02CA72 N04BC03 N06DX07 N06DX57 (ergotamin and derivates); A06AA01 A06AA51 (laxants); M03BX01 M03BX07 (muscle relexants); N05BA02 N05BA01 N05CD01 N05BA05 N05BA08 N05BA11 N05BA09 N05CD02 N05CD03 N05BA03 (long-acting benzodiazepines); N05BA12 N05CD07 N05CD05 N05BA06 N05BA56 N05BA04 N05CD06 N05CD09 N05CF02 N05CF01 N05CF03 (short- and medium-acting benzodiazepines and z-drugs); N05CM21 R06AA09 A04AB56 R06AA59 A04AB05 D04AA32 N01BX06 N05CM20 R06AA02 S01Gx16 A04Ab55 D04AA82 N05CX07 R06AA52 (other sedatives); C04AD03 C04AX21 C04AE02 N06DX13 N06BX03 (antidementia, vasodilatatives); N03AA02 N05CA24 (antiepileptics) |
| Medication: Beta-Blocker after myocardial infarction | Process indicators | Persons over 65 years with beta-blocker after myocardial infarction | Persons over 65 years after myocardial infarction | **ICD-10-GM**: I25.2 I21.- I22.-; **ATC**: C07 (beta blocker) |
| Medication: ACE-inhibitor upon hypertension and renal insufficiency | Process indicators | Persons over 65 years with ACE inhibitor, hypertensions & renal insufficiency | Persons over 65 years with hypertension and renal insufficiency | **ICD-10-GM**: I10.- I11.- I12.- I13.- I15.- (hypertension); N18.- I13.2 I13.15 N19.- (renal failure); **ATC**: C09A C09BA C09BB C09BX. |
| Medication: ACE-inhibitor upon heart failure | Process indicators | Persons over 65 years with ACE inhibitor and heart failure | Persons over 65 years with heart failure | **ICD-10-GM**: I50.1; **ATC**: C09A C09BA C09BB C09BX (ACE inhibitors) |
|  | Process indicators | Persons over 65 years with beta-blocker and bronchial asthma | Persons over 65 years with bronchial asthma | **ICD-10-GM**: J45.-; **ATC**: C07 (beta blocker) |
| Medication: Electrolyte check upon diuretics | Process indicators | Persons over 65 years with diuretics and check of electrolytes | Persons over 65 years with diuretics | **ATC**: C03 C08G C09BA C09DA B05BC C02L C07C C07D (diuretics); **GONR**: 32081 32082 32082 32082 (check of electrolytes) |
| Medication: Polypharmacy | Process indicators | Persons over 65 years with at least 5 prescription medications | Persons over 65 years with prescription medication |  |
| Ambulatory care sensitive cases | Outcome indicators | Persons with hospital admissions due to ambulatory care sensitive diagnosis | All persons | **ICD-10-GM**: I20 I25.0 I25.1 I25.5 I25.6 I25.8 I25.9 (ischaemic heart diseases); I50.- (heart failure); I05.- I06.- I08.0 I49.8 I49.9 I67.2 I67.4 I70.- I73.- I78.- I83.- I86.- I87.- I95.- I80.0 I80.80 R00.0 R00.2 R47.0 (other diseases of the circulatory system); J20.- J21.- J40.- J41.- J42.- J43.- J44.- J47.- (bronchitis & COPD); F10.- F11.- (mental and behavioural disorders due to use of alcohol or opioids); M42.- M47.- M53.- M54.- (back pain); I10.- I11.- I12.- I13.- I14.- I15 .- (hypertension); K52.2 K52.8 K52.9 K59.0 K57.- K58.- (gastroenteritis and other diseases of intestines); A01.- A02.- A04.- A05.- A07.- A08.- A09.- (intestinal infectious diseases); J10.- J11.- J13.-J14.-J15.3 J15.4 J15.7 J15.8 J16.8 J18.0 J18.1 J18.8 J18.9 (influenza and pneumonia); H66.- J01.- J02.- J03.- J06.- J31.- J32.- J35.- (Ear/nose/throat infections); F32.- F33.- (depressive disorders); E10.2 E10.3 E10.4 E10.5 E10.6 E10.8 E11.- E13.6 E13.7 E13.9 E14.- E16.2 (diabetes mellitus); M67.4 M71.3 M75.- M76.- M77.- M79.- (gonarthrosis); G56.0 M67.4 M71.3 M75.- M76.- M77.- M79.- (soft tissue disorders); F40.- F41.-F43.-F45.-F50.0F50.2 F60.- (other avoidable mental and behavioural disorders); H25.- H40.- (diseases of the eye); N30.- N34.- N39.0 (diseases of urinary system); G47.- (sleep disorders); A46.- L01.-L02.- L04.- L08.0 L08.9 L60.0 L72.1 L98.0(diseases of the skin and subcutaneous tissue); D50.- D51.- D52.- D56.-D53.1 E40.- E41.- E42.- E43.- E44.- E45.- E46.- E47.- E48.- E49.- E50.- E51.- E52.- E53.- E54.- E55.- E56.- E57.- E58.- E59.- E60.- E61.- E62.- E63.- E64.- R63.6 (malnutrition & nutritional deficiencies); K02.- K04.- K05.- K06.- K08.- K12.- K13.- (dental diseases) |
| COPD: Prevalence | Morbidity | Persons with diagnosed COPD | All persons | **ICD-10-GM**: J43.- J44.- |
| COPD: Inhalative medication | Process indicators | Persons with COPD and inhalative medication | Persons with COPD | **ICD-10-GM**: J43.- J44.-; **ATC**: R03A R03B (Inhalative medication) |
| COPD: Acute inpatient treatment | Outcome indicators | Persons with acute inpatient treatment of COPD | Persons with COPD | **ICD-10-GM**: J43.- J44.- |
| COPD: Respiratory therapy | Process indicators | Persons with COPD and respiratory therapy | Persons with COPD | **ICD-10-GM**: J43.- J44.-; **POSNR**: 302-304 501-503 601 6210 6301 6306 (respiratory therapy) |
| COPD: influenza vaccination | Process indicators | Persons with COPD and influenza vaccination | Persons with COPD | **ICD-10-GM**: J43.- J44.- Z25.1 |
| COPD: Specific beta-blocker therapy | Process indicators | Persons with COPD and specific beta-blocker therapy | Persons with COPD | **ICD-10-GM**: J43.- J44.-; **ATC**: R03AC12 R03AC13 (specific beta blocker) |
| COPD: Specific anticholinergic therapy | Process indicators | Persons with COPD and specific anticholinergic therapy | Persons with COPD | **ICD-10-GM**: J43.- J44.-; **ATC**: R03BB04 (specific anticholinergic) |
| COPD: Oral corticosteroids | Process indicators | Persons with COPD and oral corticosteroids | Persons with COPD | **ICD-10-GM**: J43.- J44.-; **ATC**: H02AB01 H02AB02 H02AB03 H02AB04 H02AB05 H02AB06 H02AB07 H02AB08 H02AB09 (corticosteroids) |
| CVD: Prevalence hypertension | Morbidity | Persons with hypertension | All persons | **ICD-10-GM**: I10.- I11.- I12.- I13.- I15.- |
| CVD: Medication for hypertension | Process indicators | Persons with hypertension and pharmacotherapy | Persons with hypertension | **ICD-10-GM**: I10.- I11.- I12.- I13.- I15.- **ATC**: C02 (antihypertensives) |
| CVD: Prevalence heart failure | Morbidity | Persons with heart failure | All persons | **ICD-10-GM**: I50.1 |
| CVD: Echocardiography upon heart failure | Process indicators | Persons with suspected heart failure and echocardiography | Persons with suspected heart failure | **ICD-10-GM**: I50.1 (suspected); **GONR**: 13545 33020 3302133022 33030 33031 |
| CVD: 12-lead ECG upon heart failure | Process indicators | Persons with suspected heart failure and 12-lead ECG | Persons with suspected heart failure | **ICD-10-GM**: I50.1 (suspected); **GONR**: 03321 04321 13250 13251 27320 27321 |
| CVD: ACE-inhibitor upon heart failure | Process indicators | Persons with heart failure treated with ACE-inhibitor or AT1-blocker | Persons with heart failure | **ICD-10-GM**: I50.1; **ATC**: C09 |
| CVD: Beta-blocker upon heart failure | Process indicators | Persons with heart failure and beta-blocker | Persons with heart failure | **ICD-10-GM**: I50.1; **ATC**: C07 |
| CVD: Anticoagulant upon atrial fibrillation and heart failure | Process indicators | Persons with heart failure and atrial fibrillation and anticoagulants | Persons with heart failure and artrial fibrillation | **ICD-10-GM**: I50.1. I48.1; **ATC**: B01AA01-B01AA08 B01AC04-B01AC08 B01AC18 B01AC22-B01AC24 B01AC34 B01AC36 B01AC56 |
| CVD: Referral to cardiologist upon heart failure | Process indicators | Persons with suspected heart failure and referral to cardiologist | Persons with suspected heart failure | **ICD-10-GM**: I50.1 (suspected); **GONR**: 13540-13542 32084. **FAGR**: 28 |
| CVD: Acute inpatient treatment of heart failure | Outcome indicators | Persons with heart failure and acute inpatient treatment of heart failure | Persons with heart failure | **ICD-10-GM**: I50.1 (hospital discharge diagnosis) |
| CVD: Apoplexy treatment in stroke unit | Process indicators | Persons with acute apoplexy and treatment in stroke-unit | Persons with acute apoplexy | **ICD-10-GM**: I61.- I63.- I64.-; **OPS**: 8981 |
| CVD: Platelet aggregation inhibitor upon stable chronic coronary heart disease | Process indicators | Persons with stable chronic coronary heart disease and platelet aggregation inhibitor | Persons with stable chronic coronary heart disease | **ICD-10-GM**: I25.-; **ATC**: B01AC06 B01AC04 |
| CVD: Statins upon coronary heart disease | Process indicators | Persons with coronary heart disease and statins | Persons with coronary heart disease | **ICD-10-GM**: I20.- I22.- I23.- I24.- I25.-; **ATC**: C10AA C10BA C10BX (statins) |
| CVD: Anti-hypertensive therapy upon coronary heart disease and hypertension | Process indicators | Persons with coronary heart disease, hypertension and anti-hypertensive therapy | Persons with coronary heart disease and hypertension | **ICD-10-GM**: I20.- I22.- I23.- I24.- I25.- (coronary heart disease); I10.- I11.- I12.- I13.- I15.- (hypertension); **ATC**: C02 C03 C07 C08 C09 (anti-hypertensive medication) |
| Dementia: Prevalence | Morbidity | Persons with dementia | All persons | **ICD-10-GM**: F00.- F01.- F02.- F03.- |
| Dementia: B12 and TSH | Process indicators | Persons with incident dementia and determination of B12 and TSH | Persons with incident dementia | **ICD-10-GM**: F00.- F01.- F02.- F03.- no such diagnosis in the prior data year; **GONR**: 32373 32101 (B12/TSH analysis) |
| T2D: Prevalence | Morbidity | Persons with type 2 diabetes mellitus | All persons | **ICD-10-GM**: E11.- |
| T2D: HbA1c | Process indicators | Persons with type 2 diabetes mellitus and check of HbA1c | Persons with type 2 diabetes mellitus | **ICD-10-GM**: E11.-; **GONR**:32094 90321 90310 90310A (HbA1c check) |
| T2D: Ophthalmological examination | Process indicators | Persons with type 2 diabetes mellitus and ophthalmological examination | Persons with type 2 diabetes mellitus | **ICD-10-GM**: E11.- **GONR**: 06210 06211 06212 (ophthalmological examination) |
| T2D: Fundus examination | Process indicators | Persons with type 2 diabetes mellitus and fundus examination | Persons with type 2 diabetes mellitus | **ICD-10-GM**: E11.-; **GONR**: 06331 06333 (fundus examination) |
| T2D: Triglycerides and cholesterol | Process indicators | Persons with type 2 diabetes mellitus and check of triglycerides, LDL- and HDL-cholesterol | Persons with type 2 diabetes mellitus | **ICD-10-GM**: E11.-; **GONR**: 32060 32061 32062 32063 (laboratory examinations) |
| T2D: Hypertension, nephropathy and ACE-inhibitor or AT1-blocker | Process indicators | Persons with type 2 diabetes mellitus, diabetic nephropathy and hypertension treated with ACE inhibitor or AT1-blocker | Persons with type 2 diabetes mellitus, diabetic nephropathy and hypertension | **ICD-10-GM**: E11.- E11.2 N08.3 N18.1 N18.2 N18.3 N18.4 N18.5 Z49.1; **ATC**: C09 |
| T2D: Serum-creatinine | Process indicators | check of serum creatinine | Persons with type 2 diabetes mellitus | **ICD-10-GM**: E11.-; **GONR**: 32066 32067. |
| Osteoarthritis: Prevalence | Morbidity | Persons with osteoarthritis | All persons | **ICD-10-GM**: M15.- M16.- M17.- M18.- M19.- |
| Osteoporosis: Prevalence | Morbidity | Persons with osteoporosis | All persons | **ICD-10-GM**: M80.- M81.- M82.- |
| Prevention: Influenza vaccination | Process indicators | Persons over 65 years with influenza vaccination | Persons over 65 years | **ICD-10-GM**: Z25.1 |
| Prevention: Mammography | Process indicators | Women between 50 and 70 years with mammography according to German cancer screening guidelines | Women between 50 and 70 years | **GONR**: 01750, Exclusion of women with **ICD-10-GM** C50.- (breast cancer) in the denominator |
| Prevention: Faecal occult blood test | Process indicators | Persons over 65 years with faecal occult blood test every 2 years according to German cancer screening guidelines | Persons over 65 years | **GONR**: 01734. Exclusion of persons with **ICD-10-GM** C18.- C19.- C20.- (colo-rectal cancer) in the denominator |
| Prevention: Men's cancer screening | Process indicators | Men over 18 years with cancer screening according to German cancer screening guidelines | Men over 18 years | **GONR**: 01731, Exclusion of persons with **ICD-10-GM** C60.- C61.- C62.- C63.- (malignant neoplasms of male genital organs) in the denominator |
| Prevention: Skin-cancer screening | Process indicators | Persons with screening for skin cancer according to German cancer screening guidelines | All persons | **GONR**: 01745, Exclusion of persons with **ICD-10-GM** C43.- C44.- (melanoma and other malignant neoplasms of skin) in the denominator |
| Depression: Prevalence | Morbidity | Persons with diagnosed depression | All persons | **ICD-10-GM**: F32.- F33.- F34.1 |
| Depression: Anti-depressive pharmacotherapy | Process indicators | Persons with diagnosed depression and anti-depressive pharmacotherapy | All persons with diagnosed depression | **ICD-10-GM**: F32.- F33.- F34.1; **ATC**: N06AA N06AB N06AF N06AG N06AX N05AN01 (anti-depressive pharmacotherapy) |

**ICD-10-GM**: International Statistical Classification of Diseases and Related Health Problems, 10th revision, German modification

**ATC**: Anatomical Therapeutic Chemical Classification System

**OPS**: German Operation and Procedure Classification System; official coding system for medical procedures in German hospitals

**GONR**: Item number (Gebührenordnungsposition) from the German Doctor's fee scale (Einheitlicher Bewertungsmaßstab, EBM)

**FAGR**: Doctor’s specialty group code according to lifelong physician identification number
