# Supplementary material for: Differences in nursing home admission between functionally defined populations in Germany and the association with quality of health care
Source: BMC Health Serv Res. 2021 Mar 2;21:190. doi: 10.1186/s12913-021-06196-8 (PMC7923327; doi:10.1186/s12913-021-06196-8)
Supplement: Supplementary file 2 — Additional file 2. [file 12913_2021_6196_MOESM2_ESM.html]

# Lists of variables per regression model

## Model 1: composition

|  |  |
| --- | --- |
| **Variable** | **Unit** |
| General practitioners | proportion (percent) |
| Ophthalmologists | proportion (percent) |
| Surgeons | proportion (percent) |
| Multidisciplinary practices | proportion (percent) |
| Therapists | proportion (percent) |
| Internists | proportion (percent) |
| Orthopaedics | proportion (percent) |
| Psychologists and psychotherapists | proportion (percent) |
| Other disciplines | proportion (percent) |
| Urologists | proportion (percent) |
| Rehabilitation facilities | proportion (percent) |
| Hospitals | proportion (percent) |
| Proportion of care-dependent persons in cluster | proportion (percent) |
| Number of providers in cluster | count, log-transformed |

## Model 2: morbidity

|  |  |
| --- | --- |
| **Variable** | **Unit** |
| Asthma: Prevalence | proportion (percent) |
| COPD: Prevalence | proportion (percent) |
| CVD: Prevalence hypertension | proportion (percent) |
| CVD: Prevalence heart failure | proportion (percent) |
| Dementia: Prevalence | proportion (percent) |
| T2D: Prevalence | proportion (percent) |
| Osteoarthritis: Prevalence | proportion (percent) |
| Osteoporosis: Prevalence | proportion (percent) |
| Depression: Prevalence | proportion (percent) |

## Model 3: process indicators

|  |  |
| --- | --- |
| **Variable** | **Unit** |
| Asthma: Spirometry | proportion (percent) |
| Asthma: Inhalative medication | proportion (percent) |
| Asthma: ICS | proportion (percent) |
| Medication: PRISCUS | proportion (percent) |
| Medication: Beta-Blocker after myocardial infarction | proportion (percent) |
| Medication: ACE-inhibitor upon hypertension and renal insufficiency | proportion (percent) |
| Medication: ACE-inhibitor upon heart failure | proportion (percent) |
| Medication: Beta-blocker upon asthma | proportion (percent) |
| Medication: Electrolyte check upon diuretics | proportion (percent) |
| Medication: Polypharmacy | proportion (percent) |
| COPD: Inhalative medication | proportion (percent) |
| COPD: Respiratory therapy | proportion (percent) |
| COPD: influenza vaccination | proportion (percent) |
| COPD: Specific beta-blocker therapy | proportion (percent) |
| COPD: Specific anticholinergic therapy | proportion (percent) |
| COPD: Oral corticosteroids | proportion (percent) |
| CVD: Medication for hypertension | proportion (percent) |
| CVD: Echocardiography upon heart failure | proportion (percent) |
| CVD: 12-lead ECG upon heart failure | proportion (percent) |
| CVD: ACE-inhibitor upon heart failure | proportion (percent) |
| CVD: Beta-blocker upon heart failure | proportion (percent) |
| CVD: Anticoagulant upon atrial fibrillation and heart failure | proportion (percent) |
| CVD: Referral to cardiologist upon heart failure | proportion (percent) |

## Model 4: continuity of care

|  |  |
| --- | --- |
| **Variable** | **Unit** |
| COC Asthma | continuous index (0–100) |
| COC COPD | continuous index (0–100) |
| COC Dementia | continuous index (0–100) |
| COC Diabetes | continuous index (0–100) |
| COC Heart Failure | continuous index (0–100) |
| SECON Asthma | continuous index (0–100) |
| SECON COPD | continuous index (0–100) |
| SECON Dementia | continuous index (0–100) |
| SECON Diabetes | continuous index (0–100) |
| SECON Heart Failure | continuous index (0–100) |
| UPC Asthma | continuous index (0–100) |
| UPC COPD | continuous index (0–100) |
| UPC Dementia | continuous index (0–100) |
| UPC Diabetes | continuous index (0–100) |
| UPC Heart Failure | continuous index (0–100) |

## odel 5: outcome indicators

|  |  |
| --- | --- |
| **Variable** | **Unit** |
| Ambulatory care sensitive cases | proportion (percent) |
| COPD: Acute inpatient treatment | proportion (percent) |
| CVD: Acute inpatient treatment of heart failure | proportion (percent) |

## Model 6: all variables

|  |  |
| --- | --- |
| **Variable** | **Unit** |
| General practitioners | proportion (percent) |
| Ophthalmologists | proportion (percent) |
| Surgeons | proportion (percent) |
| Multidisciplinary practices | proportion (percent) |
| Therapists | proportion (percent) |
| Internists | proportion (percent) |
| Orthopaedics | proportion (percent) |
| Psychologists and psychotherapists | proportion (percent) |
| Other disciplines | proportion (percent) |
| Urologists | proportion (percent) |
| Rehabilitation facilities | proportion (percent) |
| Hospitals | proportion (percent) |
| Proportion of care-dependent persons in cluster | proportion (percent) |
| Number of providers in cluster | count, log-transformed |
| Asthma: Prevalence | proportion (percent) |
| COPD: Prevalence | proportion (percent) |
| CVD: Prevalence hypertension | proportion (percent) |
| CVD: Prevalence heart failure | proportion (percent) |
| Dementia: Prevalence | proportion (percent) |
| T2D: Prevalence | proportion (percent) |
| Osteoarthritis: Prevalence | proportion (percent) |
| Osteoporosis: Prevalence | proportion (percent) |
| Depression: Prevalence | proportion (percent) |
| Asthma: Spirometry | proportion (percent) |
| Asthma: Inhalative medication | proportion (percent) |
| Asthma: ICS | proportion (percent) |
| Medication: PRISCUS | proportion (percent) |
| Medication: Beta-Blocker after myocardial infarction | proportion (percent) |
| Medication: ACE-inhibitor upon hypertension and renal insufficiency | proportion (percent) |
| Medication: ACE-inhibitor upon heart failure | proportion (percent) |
| Medication: Beta-blocker upon asthma | proportion (percent) |
| Medication: Electrolyte check upon diuretics | proportion (percent) |
| Medication: Polypharmacy | proportion (percent) |
| COPD: Inhalative medication | proportion (percent) |
| COPD: Respiratory therapy | proportion (percent) |
| COPD: influenza vaccination | proportion (percent) |
| COPD: Specific beta-blocker therapy | proportion (percent) |
| COPD: Specific anticholinergic therapy | proportion (percent) |
| COPD: Oral corticosteroids | proportion (percent) |
| CVD: Medication for hypertension | proportion (percent) |
| CVD: Echocardiography upon heart failure | proportion (percent) |
| CVD: 12-lead ECG upon heart failure | proportion (percent) |
| CVD: ACE-inhibitor upon heart failure | proportion (percent) |
| CVD: Beta-blocker upon heart failure | proportion (percent) |
| CVD: Anticoagulant upon atrial fibrillation and heart failure | proportion (percent) |
| CVD: Referral to cardiologist upon heart failure | proportion (percent) |
| COC Asthma | continuous index (0–100) |
| COC COPD | continuous index (0–100) |
| COC Dementia | continuous index (0–100) |
| COC Diabetes | continuous index (0–100) |
| COC Heart Failure | continuous index (0–100) |
| SECON Asthma | continuous index (0–100) |
| SECON COPD | continuous index (0–100) |
| SECON Dementia | continuous index (0–100) |
| SECON Diabetes | continuous index (0–100) |
| SECON Heart Failure | continuous index (0–100) |
| UPC Asthma | continuous index (0–100) |
| UPC COPD | continuous index (0–100) |
| UPC Dementia | continuous index (0–100) |
| UPC Diabetes | continuous index (0–100) |
| UPC Heart Failure | continuous index (0–100) |
| Ambulatory care sensitive cases | proportion (percent) |
| COPD: Acute inpatient treatment | proportion (percent) |
| CVD: Acute inpatient treatment of heart failure | proportion (percent) |
