# Supplementary material for: Differences in nursing home admission between functionally defined populations in Germany and the association with quality of health care
Source: BMC Health Serv Res. 2021 Mar 2;21:190. doi: 10.1186/s12913-021-06196-8 (PMC7923327; doi:10.1186/s12913-021-06196-8)
Supplement: Supplementary file 3 — Additional file 3. [file 12913_2021_6196_MOESM3_ESM.html]

Table2\_app.html.utf8.md


Summary statistics of provider cluster attributes (n=407)


|  | Value |
| --- | --- |
| **Composition: General practitioners** |  |
| Mean (SD) | 13.1 (5.5) |
| Median (Q1, Q3) | 12.0 (10.3, 14.1) |
| **Composition: Ophtalmologists** |  |
| Mean (SD) | 1.7 (0.8) |
| Median (Q1, Q3) | 1.6 (1.2, 2.0) |
| **Composition: Surgeons** |  |
| Mean (SD) | 2.2 (1.2) |
| Median (Q1, Q3) | 2.0 (1.4, 2.7) |
| **Composition: Multidisciplinary practices** |  |
| Mean (SD) | 7.7 (4.2) |
| Median (Q1, Q3) | 6.8 (5.5, 9.3) |
| **Composition: Therapists** |  |
| Mean (SD) | 45.2 (10.4) |
| Median (Q1, Q3) | 47.3 (42.5, 51.3) |
| **Composition: Internists** |  |
| Mean (SD) | 6.4 (2.5) |
| Median (Q1, Q3) | 6.2 (4.9, 7.6) |
| **Composition: Orthopedics** |  |
| Mean (SD) | 1.9 (0.9) |
| Median (Q1, Q3) | 1.8 (1.3, 2.4) |
| **Composition: Psychologists and psychotherapists** |  |
| Mean (SD) | 6.6 (2.6) |
| Median (Q1, Q3) | 6.3 (5.0, 7.9) |
| **Composition: Other disciplines** |  |
| Mean (SD) | 12.6 (4.5) |
| Median (Q1, Q3) | 11.6 (9.8, 14.6) |
| **Composition: Urologists** |  |
| Mean (SD) | 1.0 (0.6) |
| Median (Q1, Q3) | 1.0 (0.6, 1.3) |
| **Composition: Rehabilitation facilities** |  |
| Mean (SD) | 0.8 (0.6) |
| Median (Q1, Q3) | 0.8 (0.3, 1.2) |
| **Composition: Hospitals** |  |
| Mean (SD) | 0.9 (0.7) |
| Median (Q1, Q3) | 0.8 (0.5, 1.2) |
| **Composition: Proportion of care-dependent persons in cluster** |  |
| Mean (SD) | 11.1 (3.0) |
| Median (Q1, Q3) | 10.5 (8.8, 12.8) |
| **Composition: Number of providers in cluster** |  |
| Mean (SD) | 823.4 (981.5) |
| Median (Q1, Q3) | 499.0 (273.0, 998.5) |
| **No. of Comorbidities** |  |
| Mean (SD) | 1.9 (0.2) |
| Median (Q1, Q3) | 1.8 (1.7, 2.0) |
| **COC Asthma** |  |
| Mean (SD) | 69.3 (5.5) |
| Median (Q1, Q3) | 69.3 (65.7, 73.6) |
| **SECON Asthma** |  |
| Mean (SD) | 83.2 (3.9) |
| Median (Q1, Q3) | 83.5 (80.6, 86.0) |
| **UPC Asthma** |  |
| Mean (SD) | 80.7 (3.8) |
| Median (Q1, Q3) | 80.8 (78.3, 83.6) |
| **COC COPD** |  |
| Mean (SD) | 74.5 (5.3) |
| Median (Q1, Q3) | 75.0 (70.8, 78.4) |
| **SECON COPD** |  |
| Mean (SD) | 84.4 (4.0) |
| Median (Q1, Q3) | 84.7 (81.5, 87.7) |
| **UPC COPD** |  |
| Mean (SD) | 83.3 (4.0) |
| Median (Q1, Q3) | 83.7 (80.7, 86.3) |
| **COC Dementia** |  |
| Mean (SD) | 81.2 (4.1) |
| Median (Q1, Q3) | 81.6 (78.7, 84.2) |
| **SECON Dementia** |  |
| Mean (SD) | 89.4 (2.7) |
| Median (Q1, Q3) | 89.5 (87.5, 91.4) |
| **UPC Dementia** |  |
| Mean (SD) | 88.0 (2.9) |
| Median (Q1, Q3) | 88.4 (86.4, 90.1) |
| **COC Diabetes** |  |
| Mean (SD) | 78.6 (5.8) |
| Median (Q1, Q3) | 79.2 (74.7, 83.2) |
| **SECON Diabetes** |  |
| Mean (SD) | 87.9 (4.2) |
| Median (Q1, Q3) | 88.6 (85.3, 91.3) |
| **UPC Diabetes** |  |
| Mean (SD) | 85.8 (4.3) |
| Median (Q1, Q3) | 86.2 (82.8, 89.1) |
| **COC Heart Failure** |  |
| Mean (SD) | 72.1 (5.4) |
| Median (Q1, Q3) | 72.5 (68.4, 76.2) |
| **SECON Heart Failure** |  |
| Mean (SD) | 85.4 (3.7) |
| Median (Q1, Q3) | 85.6 (82.9, 88.2) |
| **UPC Heart Failure** |  |
| Mean (SD) | 82.0 (4.0) |
| Median (Q1, Q3) | 82.3 (79.3, 85.0) |
| **Asthma: Prevalence** |  |
| Mean (SD) | 3.9 (1.2) |
| Median (Q1, Q3) | 3.8 (3.2, 4.6) |
| **Asthma: Spirometry** |  |
| Mean (SD) | 36.5 (8.2) |
| Median (Q1, Q3) | 36.1 (30.5, 41.9) |
| **Asthma: Inhalative medication** |  |
| Mean (SD) | 45.8 (7.6) |
| Median (Q1, Q3) | 45.5 (40.3, 51.2) |
| **Asthma: ICS** |  |
| Mean (SD) | 26.9 (7.1) |
| Median (Q1, Q3) | 26.5 (22.1, 31.2) |
| **Medication: PRISCUS** |  |
| Mean (SD) | 20.3 (2.7) |
| Median (Q1, Q3) | 20.3 (18.2, 22.2) |
| **Medication: Beta-Blocker after myocardial infarction** |  |
| Mean (SD) | 56.2 (3.4) |
| Median (Q1, Q3) | 56.5 (54.4, 57.9) |
| **Medication: ACE-inhibitor upon hypertension and renal insufficiency a** |  |
| Mean (SD) | 47.5 (3.9) |
| Median (Q1, Q3) | 47.9 (45.3, 49.9) |
| **Medication: ACE-inhibitor upon heart failure** |  |
| Mean (SD) | 49.0 (4.1) |
| Median (Q1, Q3) | 49.1 (46.5, 51.5) |
| **Medication: Beta-blocker upon asthma** |  |
| Mean (SD) | 20.5 (4.0) |
| Median (Q1, Q3) | 20.2 (17.8, 22.9) |
| **Medication: Eletrolyte check upon diuretics** |  |
| Mean (SD) | 21.6 (5.2) |
| Median (Q1, Q3) | 21.5 (18.5, 24.9) |
| **Medication: Polypharmacy** |  |
| Mean (SD) | 33.4 (3.6) |
| Median (Q1, Q3) | 33.4 (30.9, 35.6) |
| **Ambulatory care sensitive cases** |  |
| Mean (SD) | 3.3 (0.6) |
| Median (Q1, Q3) | 3.3 (2.9, 3.7) |
| **COPD: Prevalence** |  |
| Mean (SD) | 9.2 (2.1) |
| Median (Q1, Q3) | 9.1 (7.7, 10.5) |
| **COPD: Inhalative medication** |  |
| Mean (SD) | 43.0 (4.2) |
| Median (Q1, Q3) | 43.1 (40.3, 45.9) |
| **COPD: Acute inpatient treatment** |  |
| Mean (SD) | 2.2 (0.5) |
| Median (Q1, Q3) | 2.1 (1.8, 2.5) |
| **COPD: Respiratory therapy** |  |
| Mean (SD) | 5.0 (2.3) |
| Median (Q1, Q3) | 5.1 (3.7, 6.5) |
| **COPD: influenca vaccination** |  |
| Mean (SD) | 9.3 (2.6) |
| Median (Q1, Q3) | 8.7 (7.5, 10.5) |
| **COPD: Specific beta-blocker therapy** |  |
| Mean (SD) | 34.7 (4.2) |
| Median (Q1, Q3) | 34.8 (31.7, 37.6) |
| **COPD: Specific anticholinergic therapy** |  |
| Mean (SD) | 19.7 (3.5) |
| Median (Q1, Q3) | 19.8 (17.2, 21.9) |
| **COPD: Oral corticosteroids** |  |
| Mean (SD) | 10.9 (2.8) |
| Median (Q1, Q3) | 11.0 (8.8, 12.7) |
| **CVD: Prevalence hypertension** |  |
| Mean (SD) | 62.5 (5.5) |
| Median (Q1, Q3) | 61.5 (58.3, 66.6) |
| **CVD: Medication for hypertension** |  |
| Mean (SD) | 5.4 (1.6) |
| Median (Q1, Q3) | 5.0 (4.2, 6.2) |
| **CVD: Prevalence heart failure** |  |
| Mean (SD) | 1.9 (1.1) |
| Median (Q1, Q3) | 1.7 (1.1, 2.5) |
| **CVD: Echocardiography upon heart failure** |  |
| Mean (SD) | 6.7 (6.1) |
| Median (Q1, Q3) | 4.9 (2.6, 9.1) |
| **CVD: 12-lead ECG upon heart failure** |  |
| Mean (SD) | 10.9 (7.2) |
| Median (Q1, Q3) | 9.8 (6.4, 13.7) |
| **CVD: ACE-inhibiter upon heart failure** |  |
| Mean (SD) | 58.8 (3.9) |
| Median (Q1, Q3) | 59.0 (56.3, 61.4) |
| **CVD: Beta-blocker upon heart failure** |  |
| Mean (SD) | 49.0 (4.7) |
| Median (Q1, Q3) | 49.3 (45.8, 52.0) |
| **CVD: Anticoagulant upon artrial fibrillation and heart failure** |  |
| Mean (SD) | 45.7 (3.7) |
| Median (Q1, Q3) | 45.8 (43.6, 48.1) |
| **CVD: Referral to cardiologist upon heart failure** |  |
| Mean (SD) | 11.5 (8.2) |
| Median (Q1, Q3) | 10.2 (5.8, 15.5) |
| **CVD: Acute inpatient treatment of heart failure** |  |
| Mean (SD) | 13.4 (3.3) |
| Median (Q1, Q3) | 13.0 (11.2, 15.1) |
| **CVD: Apoplexy treatment in stroke unit** |  |
| Mean (SD) | 1.6 (1.2) |
| Median (Q1, Q3) | 1.6 (0.5, 2.4) |
| **CVD: Platelet aggregation inhibitor upon stable chronic coronary heart disease** |  |
| Mean (SD) | 21.0 (4.8) |
| Median (Q1, Q3) | 21.4 (17.3, 24.7) |
| **CVD: Statins upon coronary heart disease** |  |
| Mean (SD) | 30.6 (4.8) |
| Median (Q1, Q3) | 31.3 (27.7, 34.3) |
| **CVD: Anti-hypertensive therapy upon coronary heart disease and hypertension** |  |
| Mean (SD) | 86.1 (1.5) |
| Median (Q1, Q3) | 86.3 (85.1, 87.1) |
| **Dementia: Prevalence** |  |
| Mean (SD) | 4.2 (1.1) |
| Median (Q1, Q3) | 4.0 (3.5, 4.7) |
| **Dementia: B12 and TSH** |  |
| Mean (SD) | 1.2 (1.2) |
| Median (Q1, Q3) | 1.0 (0.4, 1.7) |
| **T2D: Prevalence** |  |
| Mean (SD) | 22.1 (4.8) |
| Median (Q1, Q3) | 20.7 (18.5, 25.9) |
| **T2D: HbA1c** |  |
| Mean (SD) | 60.0 (8.0) |
| Median (Q1, Q3) | 60.7 (54.9, 66.2) |
| **T2D: Ophtalmological examination** |  |
| Mean (SD) | 29.3 (4.8) |
| Median (Q1, Q3) | 29.1 (26.2, 32.2) |
| **T2D: Fundus examination** |  |
| Mean (SD) | 16.5 (3.8) |
| Median (Q1, Q3) | 16.7 (14.6, 18.6) |
| **T2D: Triglycerides and cholesterol** |  |
| Mean (SD) | 17.5 (6.7) |
| Median (Q1, Q3) | 17.4 (12.7, 21.6) |
| **T2D: Hypertension, nepropathy and A** |  |
| Mean (SD) | 63.7 (4.4) |
| Median (Q1, Q3) | 63.8 (61.2, 66.4) |
| **T2D: Serum-creatinin** |  |
| Mean (SD) | 53.6 (7.6) |
| Median (Q1, Q3) | 53.1 (48.2, 59.2) |
| **Osteoarthritis: Prevalence** |  |
| Mean (SD) | 27.0 (3.8) |
| Median (Q1, Q3) | 26.8 (24.2, 29.3) |
| **Osteoporosis: Prevalence** |  |
| Mean (SD) | 10.9 (2.2) |
| Median (Q1, Q3) | 10.6 (9.4, 12.2) |
| **Prevention: Influenca vaccination** |  |
| Mean (SD) | 7.7 (2.7) |
| Median (Q1, Q3) | 7.0 (5.9, 8.7) |
| **Prevention: Mammography** |  |
| Mean (SD) | 2.2 (2.4) |
| Median (Q1, Q3) | 2.1 (-0.5, 4.1) |
| **Prevention: Faecal occult blood test** |  |
| Mean (SD) | 1.9 (0.5) |
| Median (Q1, Q3) | 1.9 (1.6, 2.2) |
| **Prevention: Men’s cancer screening** |  |
| Mean (SD) | 6.6 (1.5) |
| Median (Q1, Q3) | 6.6 (5.6, 7.6) |
| **Prevention: Skin-cancer screening** |  |
| Mean (SD) | 0.5 (0.3) |
| Median (Q1, Q3) | 0.5 (0.3, 0.7) |
| **Depression: Prevalence** |  |
| Mean (SD) | 11.3 (2.7) |
| Median (Q1, Q3) | 11.1 (9.7, 12.8) |
| **Depression: Anti-depressive pharmacotherapy** |  |
| Mean (SD) | 36.2 (4.1) |
| Median (Q1, Q3) | 36.1 (33.6, 38.3) |
