# Supplementary material for: Differences in nursing home admission between functionally defined populations in Germany and the association with quality of health care
Source: BMC Health Serv Res. 2021 Mar 2;21:190. doi: 10.1186/s12913-021-06196-8 (PMC7923327; doi:10.1186/s12913-021-06196-8)
Supplement: Supplementary file 4 — Additional file 4. [file 12913_2021_6196_MOESM4_ESM.html]

Proportion of nursing home admissions: results of OLS regression analyses.

|  | **Model 1** | **Model 2** | **Model 3** | **Model 4** | **Model 5** | **Model 6** | **Model 7** |
| Intercept | -138.70 | 21.85\* | 72.14 | -26.70 | 27.45\* | -241.43 | -195.13\* |
|  | [-413.96; 136.55] | [ 6.44; 37.25] | [-1.43; 145.70] | [-171.88; 118.47] | [21.95; 32.96] | [-586.44; 103.58] | [-321.66; -68.59] |
| Composition: General practitioners | 1.64 |  |  |  |  | 1.66 | 1.01\* |
|  | [ -1.11; 4.40] |  |  |  |  | [ -1.33; 4.65] | [ 0.01; 2.00] |
| Composition: Ophtalmologists | 2.86 |  |  |  |  | 0.44 |  |
|  | [ -0.53; 6.25] |  |  |  |  | [ -2.99; 3.88] |  |
| Composition: Surgeons | 1.35 |  |  |  |  | 1.74 | 0.94 |
|  | [ -1.63; 4.33] |  |  |  |  | [ -1.42; 4.90] | [ -0.38; 2.26] |
| Composition: Multidisciplinary practices | 1.57 |  |  |  |  | 1.99 | 1.34\* |
|  | [ -1.19; 4.32] |  |  |  |  | [ -0.97; 4.95] | [ 0.40; 2.27] |
| Composition: Therapists | 1.65 |  |  |  |  | 1.80 | 1.20\* |
|  | [ -1.10; 4.40] |  |  |  |  | [ -1.17; 4.76] | [ 0.29; 2.11] |
| Composition: Internists | 2.28 |  |  |  |  | 2.29 | 1.56\* |
|  | [ -0.59; 5.15] |  |  |  |  | [ -0.81; 5.38] | [ 0.45; 2.67] |
| Composition: Orthopedics | 0.12 |  |  |  |  | 2.15 | 1.48 |
|  | [ -3.09; 3.32] |  |  |  |  | [ -1.08; 5.38] | [ -0.19; 3.15] |
| Composition: Psychologists and psychotherapists | 1.16 |  |  |  |  | 2.07 | 1.39\* |
|  | [ -1.57; 3.89] |  |  |  |  | [ -0.89; 5.03] | [ 0.37; 2.41] |
| Composition: Other disciplines | 1.69 |  |  |  |  | 1.83 | 1.21\* |
|  | [ -1.08; 4.46] |  |  |  |  | [ -1.16; 4.81] | [ 0.25; 2.17] |
| Composition: Urologists | 0.77 |  |  |  |  | 0.24 |  |
|  | [ -2.77; 4.32] |  |  |  |  | [ -3.37; 3.85] |  |
| Composition: Rehabilitation facilities | 0.04 |  |  |  |  | 2.46 | 1.61\* |
|  | [ -2.75; 2.83] |  |  |  |  | [ -0.51; 5.43] | [ 0.06; 3.15] |
| Composition: Hospitals | -0.79 |  |  |  |  | 1.71 |  |
|  | [ -3.58; 2.01] |  |  |  |  | [ -1.50; 4.92] |  |
| Composition: Proportion of care-dependent persons in cluster | -0.42\* |  |  |  |  | -1.01\* | -0.98\* |
|  | [ -0.70; -0.13] |  |  |  |  | [ -1.45; -0.57] | [ -1.33; -0.64] |
| Composition: Logarithm of number of providers in cluster | 1.22\* |  |  |  |  | -0.90 |  |
|  | [ 0.21; 2.24] |  |  |  |  | [ -2.15; 0.36] |  |
| No. of Comorbidities |  | 11.03 |  |  |  | 19.80 | 13.26 |
|  |  | [-15.96; 38.01] |  |  |  | [ -14.15; 53.76] | [ -0.30; 26.81] |
| COC Asthma |  |  |  | 0.25 |  | 0.50 |  |
|  |  |  |  | [ -1.81; 2.31] |  | [ -1.74; 2.75] |  |
| SECON Asthma |  |  |  | 0.02 |  | 0.18 |  |
|  |  |  |  | [ -1.32; 1.35] |  | [ -1.26; 1.61] |  |
| UPC Asthma |  |  |  | -0.79 |  | -1.66 | -0.67 |
|  |  |  |  | [ -3.87; 2.29] |  | [ -5.03; 1.72] | [ -1.36; 0.03] |
| COC COPD |  |  |  | -7.69\* |  | -1.77 | -2.40\* |
|  |  |  |  | [ -11.83; -3.54] |  | [ -6.91; 3.37] | [ -4.54; -0.27] |
| SECON COPD |  |  |  | -1.14 |  | -1.91 | -1.50\* |
|  |  |  |  | [ -3.05; 0.77] |  | [ -4.08; 0.27] | [ -2.79; -0.20] |
| UPC COPD |  |  |  | 10.92\* |  | 4.41 | 4.78\* |
|  |  |  |  | [ 5.63; 16.22] |  | [ -1.94; 10.75] | [ 2.13; 7.43] |
| COC Dementia |  |  |  | 2.13 |  | -0.82 | -1.09 |
|  |  |  |  | [ -2.37; 6.62] |  | [ -5.40; 3.76] | [ -2.28; 0.09] |
| SECON Dementia |  |  |  | 1.40 |  | 2.75\* | 2.38\* |
|  |  |  |  | [ -0.57; 3.37] |  | [ 0.65; 4.85] | [ 0.61; 4.15] |
| UPC Dementia |  |  |  | -4.00 |  | -0.53 |  |
|  |  |  |  | [ -9.72; 1.72] |  | [ -6.39; 5.32] |  |
| COC Diabetes |  |  |  | 4.12\* |  | -0.21 |  |
|  |  |  |  | [ 0.18; 8.07] |  | [ -5.11; 4.68] |  |
| SECON Diabetes |  |  |  | 1.03 |  | 1.00 | 1.09 |
|  |  |  |  | [ -0.68; 2.74] |  | [ -1.08; 3.07] | [ -0.19; 2.38] |
| UPC Diabetes |  |  |  | -5.98\* |  | -1.07 | -1.36 |
|  |  |  |  | [ -10.48; -1.48] |  | [ -6.49; 4.35] | [ -2.73; 0.01] |
| COC Heart Failure |  |  |  | -0.31 |  | 0.17 |  |
|  |  |  |  | [ -2.51; 1.89] |  | [ -2.19; 2.53] |  |
| SECON Heart Failure |  |  |  | -0.26 |  | -0.63 |  |
|  |  |  |  | [ -1.33; 0.81] |  | [ -1.96; 0.70] |  |
| UPC Heart Failure |  |  |  | 0.63 |  | 0.68 | 0.38 |
|  |  |  |  | [ -2.19; 3.44] |  | [ -2.25; 3.61] | [ -0.06; 0.82] |
| Asthma: Prevalence |  | 0.56 |  |  |  | -0.81 | -0.77 |
|  |  | [ -0.32; 1.45] |  |  |  | [ -1.99; 0.38] | [ -1.72; 0.17] |
| Asthma: Spirometry |  |  | 0.03 |  |  | -0.09 | -0.13 |
|  |  |  | [-0.09; 0.15] |  |  | [ -0.28; 0.09] | [ -0.29; 0.04] |
| Asthma: Inhalative medication |  |  | 0.04 |  |  | 0.13 |  |
|  |  |  | [-0.14; 0.23] |  |  | [ -0.09; 0.34] |  |
| Asthma: ICS |  |  | 0.14 |  |  | 0.13 | 0.16\* |
|  |  |  | [-0.02; 0.30] |  |  | [ -0.05; 0.31] | [ 0.03; 0.29] |
| Medication: PRISCUS |  |  | -0.87\* |  |  | -1.14\* | -1.26\* |
|  |  |  | [-1.49; -0.25] |  |  | [ -1.92; -0.36] | [ -1.82; -0.71] |
| Medication: Beta-Blocker after myocardial infarction |  |  | 0.07 |  |  | 0.07 |  |
|  |  |  | [-0.36; 0.50] |  |  | [ -0.40; 0.54] |  |
| Medication: ACE-inhibitor upon hypertension and renal insufficiency a |  |  | -0.13 |  |  | -0.19 |  |
|  |  |  | [-0.60; 0.33] |  |  | [ -0.65; 0.27] |  |
| Medication: ACE-inhibitor upon heart failure |  |  | -0.06 |  |  | -0.44 | -0.56\* |
|  |  |  | [-0.60; 0.47] |  |  | [ -1.00; 0.13] | [ -0.91; -0.21] |
| Medication: Beta-blocker upon asthma |  |  | -0.32 |  |  | -0.28 |  |
|  |  |  | [-0.73; 0.10] |  |  | [ -0.72; 0.15] |  |
| Medication: Eletrolyte check upon diuretics |  |  | 0.04 |  |  | -0.07 |  |
|  |  |  | [-0.21; 0.29] |  |  | [ -0.34; 0.20] |  |
| Medication: Polypharmacy |  |  | 0.76\* |  |  | 0.93\* | 0.72\* |
|  |  |  | [ 0.24; 1.29] |  |  | [ 0.21; 1.65] | [ 0.22; 1.23] |
| Ambulatory care sensitive cases |  |  |  |  | -0.09 | -0.02 |  |
|  |  |  |  |  | [-1.81; 1.63] | [ -2.91; 2.87] |  |
| COPD: Prevalence |  | -1.07\* |  |  |  | -0.21 |  |
|  |  | [ -1.65; -0.50] |  |  |  | [ -0.96; 0.53] |  |
| COPD: Inhalative medication |  |  | 0.10 |  |  | -0.07 |  |
|  |  |  | [-0.28; 0.47] |  |  | [ -0.50; 0.36] |  |
| COPD: Acute inpatient treatment |  |  |  |  | -2.28\* | 2.81\* | 2.17\* |
|  |  |  |  |  | [-4.33; -0.23] | [ 0.06; 5.57] | [ 0.32; 4.02] |
| COPD: Respiratory therapy |  |  | -0.14 |  |  | 0.06 |  |
|  |  |  | [-0.60; 0.33] |  |  | [ -0.53; 0.66] |  |
| COPD: influenca vaccination |  |  | 1.01 |  |  | 0.38 |  |
|  |  |  | [-0.55; 2.56] |  |  | [ -1.17; 1.92] |  |
| COPD: Specific beta-blocker therapy |  |  | -0.22 |  |  | -0.13 |  |
|  |  |  | [-0.61; 0.18] |  |  | [ -0.57; 0.31] |  |
| COPD: Specific anticholinergic therapy |  |  | 0.03 |  |  | 0.01 |  |
|  |  |  | [-0.31; 0.37] |  |  | [ -0.35; 0.37] |  |
| COPD: Oral corticosteroids |  |  | -0.23 |  |  | -0.21 |  |
|  |  |  | [-0.73; 0.27] |  |  | [ -0.76; 0.33] |  |
| CVD: Prevalence hypertension |  | -0.20 |  |  |  | -0.44 | -0.41 |
|  |  | [ -0.63; 0.23] |  |  |  | [ -1.02; 0.13] | [ -0.84; 0.01] |
| CVD: Medication for hypertension |  |  | 0.24 |  |  | 0.01 |  |
|  |  |  | [-0.48; 0.96] |  |  | [ -0.80; 0.82] |  |
| CVD: Prevalence heart failure |  | -1.47\* |  |  |  | -2.12\* | -1.92\* |
|  |  | [ -2.48; -0.47] |  |  |  | [ -3.49; -0.75] | [ -3.01; -0.82] |
| CVD: Echocardiography upon heart failure |  |  | -0.24\* |  |  | -0.15\* | -0.12 |
|  |  |  | [-0.38; -0.10] |  |  | [ -0.29; -0.01] | [ -0.25; 0.00] |
| CVD: 12-lead ECG upon heart failure |  |  | -0.06 |  |  | -0.05 | -0.10 |
|  |  |  | [-0.19; 0.07] |  |  | [ -0.18; 0.08] | [ -0.21; 0.02] |
| CVD: ACE-inhibiter upon heart failure |  |  | -0.08 |  |  | 0.48 | 0.80\* |
|  |  |  | [-0.56; 0.41] |  |  | [ -0.05; 1.02] | [ 0.38; 1.23] |
| CVD: Beta-blocker upon heart failure |  |  | 0.23 |  |  | 0.26 |  |
|  |  |  | [-0.13; 0.58] |  |  | [ -0.11; 0.63] |  |
| CVD: Anticoagulant upon artrial fibrillation and heart failure |  |  | -0.29 |  |  | -0.30 | -0.24 |
|  |  |  | [-0.68; 0.09] |  |  | [ -0.70; 0.10] | [ -0.56; 0.09] |
| CVD: Referral to cardiologist upon heart failure |  |  | -0.05 |  |  | -0.00 |  |
|  |  |  | [-0.14; 0.04] |  |  | [ -0.10; 0.09] |  |
| CVD: Acute inpatient treatment of heart failure |  |  |  |  | 0.18 | -0.37 | -0.33\* |
|  |  |  |  |  | [-0.10; 0.46] | [ -0.76; 0.01] | [ -0.65; -0.02] |
| CVD: Apoplexy treatment in stroke unit |  |  | 0.47 |  |  | 0.68 | 0.69 |
|  |  |  | [-0.30; 1.25] |  |  | [ -0.12; 1.48] | [ -0.01; 1.39] |
| CVD: Platelet aggregation inhibitor upon stable chronic coronary heart disease |  |  | -0.10 |  |  | -0.01 |  |
|  |  |  | [-0.44; 0.25] |  |  | [ -0.38; 0.35] |  |
| CVD: Statins upon coronary heart disease |  |  | -0.18 |  |  | -0.57\* | -0.47\* |
|  |  |  | [-0.49; 0.14] |  |  | [ -0.92; -0.21] | [ -0.72; -0.22] |
| CVD: Anti-hypertensive therapy upon coronary heart disease and hypertension |  |  | 0.00 |  |  | 0.64 |  |
|  |  |  | [-1.01; 1.02] |  |  | [ -0.45; 1.72] |  |
| Dementia: Prevalence |  | 1.46\* |  |  |  | 2.02\* | 1.79\* |
|  |  | [ 0.23; 2.70] |  |  |  | [ 0.61; 3.44] | [ 0.68; 2.90] |
| Dementia: B12 and TSH |  |  | 0.67 |  |  | 0.50 |  |
|  |  |  | [-0.07; 1.41] |  |  | [ -0.31; 1.31] |  |
| T2D: Prevalence |  | 0.29 |  |  |  | -0.15 |  |
|  |  | [ -0.18; 0.77] |  |  |  | [ -0.79; 0.49] |  |
| T2D: HbA1c |  |  | -0.15\* |  |  | -0.19\* | -0.23\* |
|  |  |  | [-0.29; -0.00] |  |  | [ -0.37; -0.01] | [ -0.37; -0.10] |
| T2D: Ophtalmological examination |  |  | 0.03 |  |  | 0.14 |  |
|  |  |  | [-0.20; 0.26] |  |  | [ -0.11; 0.39] |  |
| T2D: Fundus examination |  |  | -0.23 |  |  | -0.09 |  |
|  |  |  | [-0.52; 0.05] |  |  | [ -0.40; 0.22] |  |
| T2D: Triglycerides and cholesterol |  |  | -0.19\* |  |  | -0.13 | -0.18\* |
|  |  |  | [-0.32; -0.07] |  |  | [ -0.26; 0.01] | [ -0.29; -0.06] |
| T2D: Hypertension, nepropathy and A |  |  | -0.14 |  |  | -0.42\* | -0.53\* |
|  |  |  | [-0.44; 0.17] |  |  | [ -0.74; -0.11] | [ -0.79; -0.27] |
| T2D: Serum-creatinin |  |  | -0.03 |  |  | 0.11 | 0.10 |
|  |  |  | [-0.20; 0.14] |  |  | [ -0.09; 0.30] | [ -0.04; 0.25] |
| Osteoarthritis: Prevalence |  | 0.01 |  |  |  | -0.03 |  |
|  |  | [ -0.41; 0.42] |  |  |  | [ -0.57; 0.50] |  |
| Osteoporosis: Prevalence |  | -0.17 |  |  |  | -0.45 | -0.49 |
|  |  | [ -0.72; 0.38] |  |  |  | [ -1.07; 0.18] | [ -0.99; 0.00] |
| Prevention: Influenca vaccination |  |  | -0.99 |  |  | -0.74 |  |
|  |  |  | [-2.74; 0.77] |  |  | [ -2.61; 1.13] |  |
| Prevention: Mammography |  |  | -0.36 |  |  | -0.25 |  |
|  |  |  | [-0.83; 0.11] |  |  | [ -0.81; 0.31] |  |
| Prevention: Faecal occult blood test |  |  | 0.22 |  |  | 0.07 |  |
|  |  |  | [-2.07; 2.51] |  |  | [ -2.32; 2.46] |  |
| Prevention: Men's cancer screening |  |  | -0.34 |  |  | -0.43 | -0.59\* |
|  |  |  | [-1.03; 0.35] |  |  | [ -1.13; 0.27] | [ -1.17; -0.00] |
| Prevention: Skin-cancer screening |  |  | 1.19 |  |  | -1.06 |  |
|  |  |  | [-2.28; 4.65] |  |  | [ -4.70; 2.58] |  |
| Depression: Prevalence |  | -0.54\* |  |  |  | -0.41 |  |
|  |  | [ -0.99; -0.09] |  |  |  | [ -1.09; 0.27] |  |
| Depression: Anti-depressive pharmacotherapy |  |  | -0.18 |  |  | -0.25 |  |
|  |  |  | [-0.44; 0.08] |  |  | [ -0.55; 0.04] |  |
| No. of variables | 14 | 10 | 40 | 15 | 3 | 82 | 34 |
| Num. obs. | 407 | 407 | 407 | 407 | 407 | 407 | 407 |
| R2 | 0.11 | 0.20 | 0.33 | 0.21 | 0.02 | 0.49 | 0.46 |
| Adj. R2 | 0.08 | 0.18 | 0.26 | 0.18 | 0.01 | 0.36 | 0.40 |
| AIC | 3058 | 3010 | 2996 | 3013 | 3078 | 2967 | 2912 |
| \* 0 outside the confidence interval. | | | | | | | | |
