# Supplementary material for: Differences in nursing home admission between functionally defined populations in Germany and the association with quality of health care
Source: BMC Health Serv Res. 2021 Mar 2;21:190. doi: 10.1186/s12913-021-06196-8 (PMC7923327; doi:10.1186/s12913-021-06196-8)
Supplement: Supplementary file 5 — Additional file 5. [file 12913_2021_6196_MOESM5_ESM.html]

Average time (in quarters) until nursing home admission: results of OLS regression analyses.

|  | **Model 1** | **Model 2** | **Model 3** | **Model 4** | **Model 5** | **Model 6** | **Model 7** |
| Intercept | 32.66 | 6.31 | -22.42 | -18.46 | 17.63\* | -130.13 | -45.12 |
|  | [-143.84; 209.16] | [ -5.24; 17.86] | [-72.64; 27.80] | [-124.69; 87.76] | [13.68; 21.57] | [-359.42; 99.16] | [-102.49; 12.24] |
| Composition: General practitioners | -0.05 |  |  |  |  | 0.92 | 0.26 |
|  | [ -1.81; 1.72] |  |  |  |  | [ -1.07; 2.90] | [ -0.07; 0.60] |
| Composition: Ophtalmologists | -0.03 |  |  |  |  | 0.13 |  |
|  | [ -2.21; 2.14] |  |  |  |  | [ -2.15; 2.41] |  |
| Composition: Surgeons | -0.17 |  |  |  |  | 1.57 | 0.68\* |
|  | [ -2.08; 1.74] |  |  |  |  | [ -0.53; 3.67] | [ 0.05; 1.31] |
| Composition: Multidisciplinary practices | -0.42 |  |  |  |  | 1.12 | 0.42\* |
|  | [ -2.19; 1.34] |  |  |  |  | [ -0.85; 3.09] | [ 0.18; 0.66] |
| Composition: Therapists | -0.29 |  |  |  |  | 1.10 | 0.42\* |
|  | [ -2.05; 1.48] |  |  |  |  | [ -0.87; 3.07] | [ 0.19; 0.66] |
| Composition: Internists | -1.20 |  |  |  |  | 0.71 |  |
|  | [ -3.04; 0.64] |  |  |  |  | [ -1.35; 2.76] |  |
| Composition: Orthopedics | -1.04 |  |  |  |  | 1.04 |  |
|  | [ -3.10; 1.01] |  |  |  |  | [ -1.10; 3.19] |  |
| Composition: Psychologists and psychotherapists | -0.87 |  |  |  |  | 1.11 | 0.44\* |
|  | [ -2.62; 0.89] |  |  |  |  | [ -0.85; 3.08] | [ 0.10; 0.79] |
| Composition: Other disciplines | -0.24 |  |  |  |  | 1.21 | 0.49\* |
|  | [ -2.02; 1.54] |  |  |  |  | [ -0.77; 3.20] | [ 0.22; 0.77] |
| Composition: Urologists | 0.67 |  |  |  |  | 2.18 | 1.43\* |
|  | [ -1.61; 2.94] |  |  |  |  | [ -0.22; 4.58] | [ 0.17; 2.69] |
| Composition: Rehabilitation facilities | 0.62 |  |  |  |  | 1.68 | 1.27\* |
|  | [ -1.17; 2.41] |  |  |  |  | [ -0.29; 3.66] | [ 0.37; 2.17] |
| Composition: Hospitals | -0.06 |  |  |  |  | 0.67 |  |
|  | [ -1.85; 1.73] |  |  |  |  | [ -1.46; 2.80] |  |
| Composition: Proportion of care-dependent persons in cluster | 0.33\* |  |  |  |  | 0.50\* | 0.54\* |
|  | [ 0.15; 0.51] |  |  |  |  | [ 0.21; 0.79] | [ 0.32; 0.76] |
| Composition: Logarithm of number of providers in cluster | 2.13\* |  |  |  |  | 1.71\* | 1.76\* |
|  | [ 1.48; 2.78] |  |  |  |  | [ 0.87; 2.54] | [ 1.08; 2.45] |
| No. of Comorbidities |  | -20.15 |  |  |  | -26.54\* | -18.14\* |
|  |  | [-40.38; 0.09] |  |  |  | [ -49.11; -3.98] | [ -26.82; -9.47] |
| COC Asthma |  |  |  | 0.67 |  | -1.06 | -1.22\* |
|  |  |  |  | [ -0.84; 2.17] |  | [ -2.55; 0.43] | [ -2.27; -0.17] |
| SECON Asthma |  |  |  | -0.95 |  | -0.34 |  |
|  |  |  |  | [ -1.93; 0.03] |  | [ -1.29; 0.62] |  |
| UPC Asthma |  |  |  | 0.34 |  | 2.15 | 2.16\* |
|  |  |  |  | [ -1.92; 2.59] |  | [ -0.09; 4.39] | [ 0.59; 3.73] |
| COC COPD |  |  |  | -7.51\* |  | -0.50 |  |
|  |  |  |  | [ -10.54; -4.47] |  | [ -3.91; 2.92] |  |
| SECON COPD |  |  |  | 2.10\* |  | 1.32 | 1.04\* |
|  |  |  |  | [ 0.70; 3.49] |  | [ -0.12; 2.77] | [ 0.15; 1.94] |
| UPC COPD |  |  |  | 7.33\* |  | -1.22 | -1.59\* |
|  |  |  |  | [ 3.46; 11.20] |  | [ -5.44; 2.99] | [ -2.63; -0.56] |
| COC Dementia |  |  |  | -1.49 |  | -0.10 |  |
|  |  |  |  | [ -4.78; 1.79] |  | [ -3.14; 2.94] |  |
| SECON Dementia |  |  |  | 2.61\* |  | 0.45 |  |
|  |  |  |  | [ 1.17; 4.05] |  | [ -0.94; 1.85] |  |
| UPC Dementia |  |  |  | -0.53 |  | -0.23 |  |
|  |  |  |  | [ -4.71; 3.66] |  | [ -4.13; 3.66] |  |
| COC Diabetes |  |  |  | 6.16\* |  | 1.35 | 0.60 |
|  |  |  |  | [ 3.27; 9.04] |  | [ -1.91; 4.60] | [ -0.17; 1.37] |
| SECON Diabetes |  |  |  | -2.81\* |  | -1.05 | -0.75 |
|  |  |  |  | [ -4.06; -1.56] |  | [ -2.43; 0.32] | [ -1.71; 0.22] |
| UPC Diabetes |  |  |  | -5.33\* |  | -0.70 |  |
|  |  |  |  | [ -8.62; -2.04] |  | [ -4.31; 2.90] |  |
| COC Heart Failure |  |  |  | 0.40 |  | -0.28 |  |
|  |  |  |  | [ -1.22; 2.01] |  | [ -1.85; 1.28] |  |
| SECON Heart Failure |  |  |  | -0.66 |  | -0.21 |  |
|  |  |  |  | [ -1.45; 0.12] |  | [ -1.10; 0.67] |  |
| UPC Heart Failure |  |  |  | -0.06 |  | 0.63 |  |
|  |  |  |  | [ -2.12; 2.00] |  | [ -1.32; 2.58] |  |
| Asthma: Prevalence |  | -0.02 |  |  |  | -0.08 |  |
|  |  | [ -0.68; 0.65] |  |  |  | [ -0.87; 0.71] |  |
| Asthma: Spirometry |  |  | -0.01 |  |  | 0.01 |  |
|  |  |  | [ -0.10; 0.07] |  |  | [ -0.11; 0.13] |  |
| Asthma: Inhalative medication |  |  | 0.01 |  |  | 0.04 |  |
|  |  |  | [ -0.12; 0.13] |  |  | [ -0.10; 0.18] |  |
| Asthma: ICS |  |  | -0.04 |  |  | -0.00 |  |
|  |  |  | [ -0.15; 0.06] |  |  | [ -0.12; 0.12] |  |
| Medication: PRISCUS |  |  | -0.46\* |  |  | -0.37 |  |
|  |  |  | [ -0.89; -0.04] |  |  | [ -0.89; 0.15] |  |
| Medication: Beta-Blocker after myocardial infarction |  |  | -0.08 |  |  | -0.07 |  |
|  |  |  | [ -0.37; 0.22] |  |  | [ -0.39; 0.24] |  |
| Medication: ACE-inhibitor upon hypertension and renal insufficiency a |  |  | -0.30 |  |  | -0.15 |  |
|  |  |  | [ -0.61; 0.02] |  |  | [ -0.46; 0.16] |  |
| Medication: ACE-inhibitor upon heart failure |  |  | -0.17 |  |  | -0.19 | -0.36\* |
|  |  |  | [ -0.53; 0.20] |  |  | [ -0.57; 0.18] | [ -0.58; -0.14] |
| Medication: Beta-blocker upon asthma |  |  | -0.22 |  |  | -0.02 |  |
|  |  |  | [ -0.50; 0.06] |  |  | [ -0.31; 0.27] |  |
| Medication: Eletrolyte check upon diuretics |  |  | -0.11 |  |  | -0.07 |  |
|  |  |  | [ -0.28; 0.06] |  |  | [ -0.25; 0.11] |  |
| Medication: Polypharmacy |  |  | 0.56\* |  |  | 0.62\* | 0.34\* |
|  |  |  | [ 0.21; 0.92] |  |  | [ 0.15; 1.10] | [ 0.10; 0.59] |
| Ambulatory care sensitive cases |  |  |  |  | 0.48 | -2.13\* | -1.87\* |
|  |  |  |  |  | [-0.75; 1.72] | [ -4.05; -0.21] | [ -3.27; -0.47] |
| COPD: Prevalence |  | -0.08 |  |  |  | 0.48 | 0.32 |
|  |  | [ -0.51; 0.35] |  |  |  | [ -0.01; 0.98] | [ -0.04; 0.68] |
| COPD: Inhalative medication |  |  | -0.20 |  |  | -0.35\* | -0.33\* |
|  |  |  | [ -0.45; 0.06] |  |  | [ -0.63; -0.07] | [ -0.51; -0.15] |
| COPD: Acute inpatient treatment |  |  |  |  | -0.74 | 1.11 | 1.54 |
|  |  |  |  |  | [-2.21; 0.73] | [ -0.72; 2.94] | [ -0.01; 3.08] |
| COPD: Respiratory therapy |  |  | -0.08 |  |  | -0.39 | -0.38\* |
|  |  |  | [ -0.39; 0.24] |  |  | [ -0.79; 0.00] | [ -0.68; -0.07] |
| COPD: influenca vaccination |  |  | 0.23 |  |  | 0.37 |  |
|  |  |  | [ -0.84; 1.29] |  |  | [ -0.65; 1.40] |  |
| COPD: Specific beta-blocker therapy |  |  | 0.12 |  |  | -0.11 |  |
|  |  |  | [ -0.15; 0.39] |  |  | [ -0.41; 0.18] |  |
| COPD: Specific anticholinergic therapy |  |  | 0.04 |  |  | 0.08 |  |
|  |  |  | [ -0.19; 0.27] |  |  | [ -0.16; 0.32] |  |
| COPD: Oral corticosteroids |  |  | 0.07 |  |  | 0.03 |  |
|  |  |  | [ -0.27; 0.41] |  |  | [ -0.33; 0.39] |  |
| CVD: Prevalence hypertension |  | 0.44\* |  |  |  | 0.27 | 0.38\* |
|  |  | [ 0.12; 0.76] |  |  |  | [ -0.11; 0.66] | [ 0.11; 0.64] |
| CVD: Medication for hypertension |  |  | -0.63\* |  |  | -0.49 | -0.32 |
|  |  |  | [ -1.13; -0.14] |  |  | [ -1.03; 0.05] | [ -0.78; 0.13] |
| CVD: Prevalence heart failure |  | 0.73 |  |  |  | 0.21 |  |
|  |  | [ -0.03; 1.48] |  |  |  | [ -0.70; 1.12] |  |
| CVD: Echocardiography upon heart failure |  |  | 0.01 |  |  | -0.00 |  |
|  |  |  | [ -0.09; 0.10] |  |  | [ -0.10; 0.09] |  |
| CVD: 12-lead ECG upon heart failure |  |  | 0.00 |  |  | -0.03 |  |
|  |  |  | [ -0.08; 0.09] |  |  | [ -0.11; 0.06] |  |
| CVD: ACE-inhibiter upon heart failure |  |  | 0.21 |  |  | 0.20 | 0.25\* |
|  |  |  | [ -0.12; 0.54] |  |  | [ -0.15; 0.56] | [ 0.02; 0.49] |
| CVD: Beta-blocker upon heart failure |  |  | -0.10 |  |  | 0.00 |  |
|  |  |  | [ -0.34; 0.15] |  |  | [ -0.25; 0.25] |  |
| CVD: Anticoagulant upon artrial fibrillation and heart failure |  |  | 0.14 |  |  | 0.08 |  |
|  |  |  | [ -0.12; 0.41] |  |  | [ -0.18; 0.35] |  |
| CVD: Referral to cardiologist upon heart failure |  |  | 0.07\* |  |  | 0.04 |  |
|  |  |  | [ 0.00; 0.13] |  |  | [ -0.03; 0.10] |  |
| CVD: Acute inpatient treatment of heart failure |  |  |  |  | -0.07 | -0.04 |  |
|  |  |  |  |  | [-0.28; 0.13] | [ -0.29; 0.22] |  |
| CVD: Apoplexy treatment in stroke unit |  |  | 0.20 |  |  | 0.10 |  |
|  |  |  | [ -0.32; 0.73] |  |  | [ -0.43; 0.64] |  |
| CVD: Platelet aggregation inhibitor upon stable chronic coronary heart disease |  |  | 0.01 |  |  | -0.00 |  |
|  |  |  | [ -0.22; 0.25] |  |  | [ -0.25; 0.24] |  |
| CVD: Statins upon coronary heart disease |  |  | -0.11 |  |  | -0.16 |  |
|  |  |  | [ -0.32; 0.11] |  |  | [ -0.39; 0.08] |  |
| CVD: Anti-hypertensive therapy upon coronary heart disease and hypertension |  |  | 0.83\* |  |  | 0.53 |  |
|  |  |  | [ 0.14; 1.52] |  |  | [ -0.19; 1.25] |  |
| Dementia: Prevalence |  | -0.52 |  |  |  | 0.17 |  |
|  |  | [ -1.45; 0.40] |  |  |  | [ -0.77; 1.11] |  |
| Dementia: B12 and TSH |  |  | 0.24 |  |  | -0.34 |  |
|  |  |  | [ -0.27; 0.75] |  |  | [ -0.88; 0.20] |  |
| T2D: Prevalence |  | 0.39\* |  |  |  | 0.10 |  |
|  |  | [ 0.03; 0.74] |  |  |  | [ -0.32; 0.52] |  |
| T2D: HbA1c |  |  | -0.22\* |  |  | -0.28\* | -0.29\* |
|  |  |  | [ -0.32; -0.12] |  |  | [ -0.40; -0.17] | [ -0.38; -0.21] |
| T2D: Ophtalmological examination |  |  | 0.20\* |  |  | 0.24\* | 0.20\* |
|  |  |  | [ 0.05; 0.36] |  |  | [ 0.07; 0.40] | [ 0.06; 0.33] |
| T2D: Fundus examination |  |  | -0.37\* |  |  | -0.35\* | -0.36\* |
|  |  |  | [ -0.56; -0.18] |  |  | [ -0.56; -0.15] | [ -0.52; -0.20] |
| T2D: Triglycerides and cholesterol |  |  | 0.01 |  |  | 0.02 |  |
|  |  |  | [ -0.08; 0.09] |  |  | [ -0.07; 0.11] |  |
| T2D: Hypertension, nepropathy and A |  |  | -0.06 |  |  | -0.04 |  |
|  |  |  | [ -0.27; 0.15] |  |  | [ -0.25; 0.17] |  |
| T2D: Serum-creatinin |  |  | 0.31\* |  |  | 0.23\* | 0.22\* |
|  |  |  | [ 0.19; 0.42] |  |  | [ 0.10; 0.36] | [ 0.13; 0.31] |
| Osteoarthritis: Prevalence |  | 0.23 |  |  |  | 0.32 | 0.23 |
|  |  | [ -0.09; 0.54] |  |  |  | [ -0.03; 0.67] | [ -0.00; 0.47] |
| Osteoporosis: Prevalence |  | 0.02 |  |  |  | 0.13 |  |
|  |  | [ -0.39; 0.43] |  |  |  | [ -0.29; 0.54] |  |
| Prevention: Influenca vaccination |  |  | -0.47 |  |  | -0.19 |  |
|  |  |  | [ -1.67; 0.73] |  |  | [ -1.43; 1.06] |  |
| Prevention: Mammography |  |  | -0.82\* |  |  | -0.96\* | -0.92\* |
|  |  |  | [ -1.14; -0.51] |  |  | [ -1.33; -0.59] | [ -1.21; -0.63] |
| Prevention: Faecal occult blood test |  |  | -1.78\* |  |  | -1.02 | -1.05 |
|  |  |  | [ -3.34; -0.21] |  |  | [ -2.61; 0.57] | [ -2.38; 0.29] |
| Prevention: Men's cancer screening |  |  | -0.03 |  |  | -0.23 |  |
|  |  |  | [ -0.50; 0.44] |  |  | [ -0.69; 0.24] |  |
| Prevention: Skin-cancer screening |  |  | 0.01 |  |  | -0.32 |  |
|  |  |  | [ -2.35; 2.38] |  |  | [ -2.74; 2.10] |  |
| Depression: Prevalence |  | 0.60\* |  |  |  | 0.19 |  |
|  |  | [ 0.26; 0.94] |  |  |  | [ -0.26; 0.65] |  |
| Depression: Anti-depressive pharmacotherapy |  |  | -0.17 |  |  | -0.03 |  |
|  |  |  | [ -0.35; 0.01] |  |  | [ -0.23; 0.17] |  |
| No. of variables | 14 | 10 | 40 | 15 | 3 | 82 | 34 |
| Num. obs. | 407 | 407 | 407 | 407 | 407 | 407 | 407 |
| R2 | 0.28 | 0.11 | 0.38 | 0.16 | 0.00 | 0.56 | 0.53 |
| Adj. R2 | 0.25 | 0.09 | 0.32 | 0.13 | -0.00 | 0.45 | 0.49 |
| AIC | 2697 | 2776 | 2686 | 2759 | 2807 | 2634 | 2563 |
| \* 0 outside the confidence interval. | | | | | | | | |
